# Single-cell expression profile of Drosophila ovarian follicle stem cells illuminates spatial differentiation in the germarium

**DOI:** 10.1186/s12915-023-01636-9

**Published:** 2023-06-20

**Authors:** Zhi Dong, Lan Pang, Zhiguo Liu, Yifeng Sheng, Xiaoping Li, Xavier Thibault, Amy Reilein, Daniel Kalderon, Jianhua Huang

**Affiliations:** 1 Institute of Insect Sciences, Ministry of Agriculture Key Lab of Molecular Biology of Crop Pathogens and Insect Pests, College of Agriculture and Biotechnology, Zhejiang University, Hangzhou, 310058 China; 2 Department of Hepatic Surgery and Liver Transplantation Center of the Third Affiliated Hospital, Organ Transplantation Institute, Sun Yat-Sen University, Guangzhou, 510630 Guangdong China; 3 Department of Biological Sciences, Columbia University, New York, NY USA

**Keywords:** Drosophila, Adult stem cells, Follicle stem cells, Ovary, Single-cell RNA sequencing

## Abstract

**Background:**

How stem cell populations are organized and regulated within adult tissues is important for understanding cancer origins and for developing cell replacement strategies. Paradigms such as mammalian gut stem cells and Drosophila ovarian follicle stem cells (FSC) are characterized by population asymmetry, in which stem cell division and differentiation are separately regulated processes. These stem cells behave stochastically regarding their contributions to derivative cells and also exhibit dynamic spatial heterogeneity. Drosophila FSCs provide an excellent model for understanding how a community of active stem cells maintained by population asymmetry is regulated. Here, we use single-cell RNA sequencing to profile the gene expression patterns of FSCs and their immediate derivatives to investigate heterogeneity within the stem cell population and changes associated with differentiation.

**Results:**

We describe single-cell RNA sequencing studies of a pre-sorted population of cells that include FSCs and the neighboring cell types, escort cells (ECs) and follicle cells (FCs), which they support. Cell-type assignment relies on anterior–posterior (AP) location within the germarium. We clarify the previously determined location of FSCs and use spatially targeted lineage studies as further confirmation. The scRNA profiles among four clusters are consistent with an AP progression from anterior ECs through posterior ECs and then FSCs, to early FCs. The relative proportion of EC and FSC clusters are in good agreement with the prevalence of those cell types in a germarium. Several genes with graded profiles from ECs to FCs are highlighted as candidate effectors of the inverse gradients of the two principal signaling pathways, Wnt and JAK-STAT, that guide FSC differentiation and division.

**Conclusions:**

Our data establishes an important resource of scRNA-seq profiles for FSCs and their immediate derivatives that is based on precise spatial location and functionally established stem cell identity, and facilitates future genetic investigation of regulatory interactions guiding FSC behavior.

**Supplementary Information:**

The online version contains supplementary material available at 10.1186/s12915-023-01636-9.

## Background

Adult stem cells provide a lifelong source of specific differentiated cells, necessitated by physiological turnover or changing environmental conditions [1–3]. The study of adult stem cells is revealing that this task can be accomplished in a variety of ways. Importantly, it is often a shared task. In such paradigms, often termed population asymmetry, individual stem cells make stochastic decisions about cell division and differentiation [2–8]. Division and differentiation are independent processes in the three best-studied paradigms [9–11] and are balanced at the community level. The stochastic variation in behavior of individual stem cells may be compounded further by systematic heterogeneity based on precise stem cell location [2, 12]. For example, within a population of about 16 follicle stem cells (FSCs) in a Drosophila germarium, roughly half are in a posterior ring. Only posterior FSCs can directly become proliferative follicle cells (FCs) and they also divide much faster than their anterior FSC neighbors, which can directly become quiescent escort cells (ECs) [13]. Posterior and anterior FSCs nevertheless form a single community because they can exchange positions [13]. In other paradigms, it has also often been observed that stem cell derivatives can revert to stem cell status under physiological or stress conditions [14–16]. All of these plastic properties, including variable stem cell lifetimes, differ substantially from the original concept, still relevant to some paradigms, of each stem cell behaving the same way—dividing with irreversible asymmetric outcomes for the two daughters and exhibiting exceptional longevity [1–3, 7].

Many aspects of the status of a cell can be captured by a detailed rendition of its pattern of RNA expression, so understanding of adult stem cell biology surely benefits from such information, typically captured by single-cell RNA (scRNA) sequencing. However, adult stem cell paradigms that exhibit stochastic and heterogeneous behaviors, with derivative cells that are necessarily initially very similar to stem cells, present a significant challenge to categorization of stem cells and their immediate derivatives according to scRNA profiles. These profiles cannot be expected to reveal stem cells as a highly distinct group and certainly cannot suffice to define stem cells. Instead, they must be related carefully to the results of functional tests that define stem cells through their behavior [2]. Drosophila FSCs present a particularly attractive paradigm for such analyses because their behavior is complex but has been studied carefully, along with extensive investigation of key regulatory niche signals [2, 9, 13, 17, 18]. Here, we present a scRNA study of somatic ECs, FSCs, and early FCs within the Drosophila germarium and we relate those studies to functional tests of FSC behavior based on location. The resulting expression profiles reveal candidate positionally regulated effectors of FSC division and differentiation.

## Results

### Location of FSCs

**Fig. 1:**
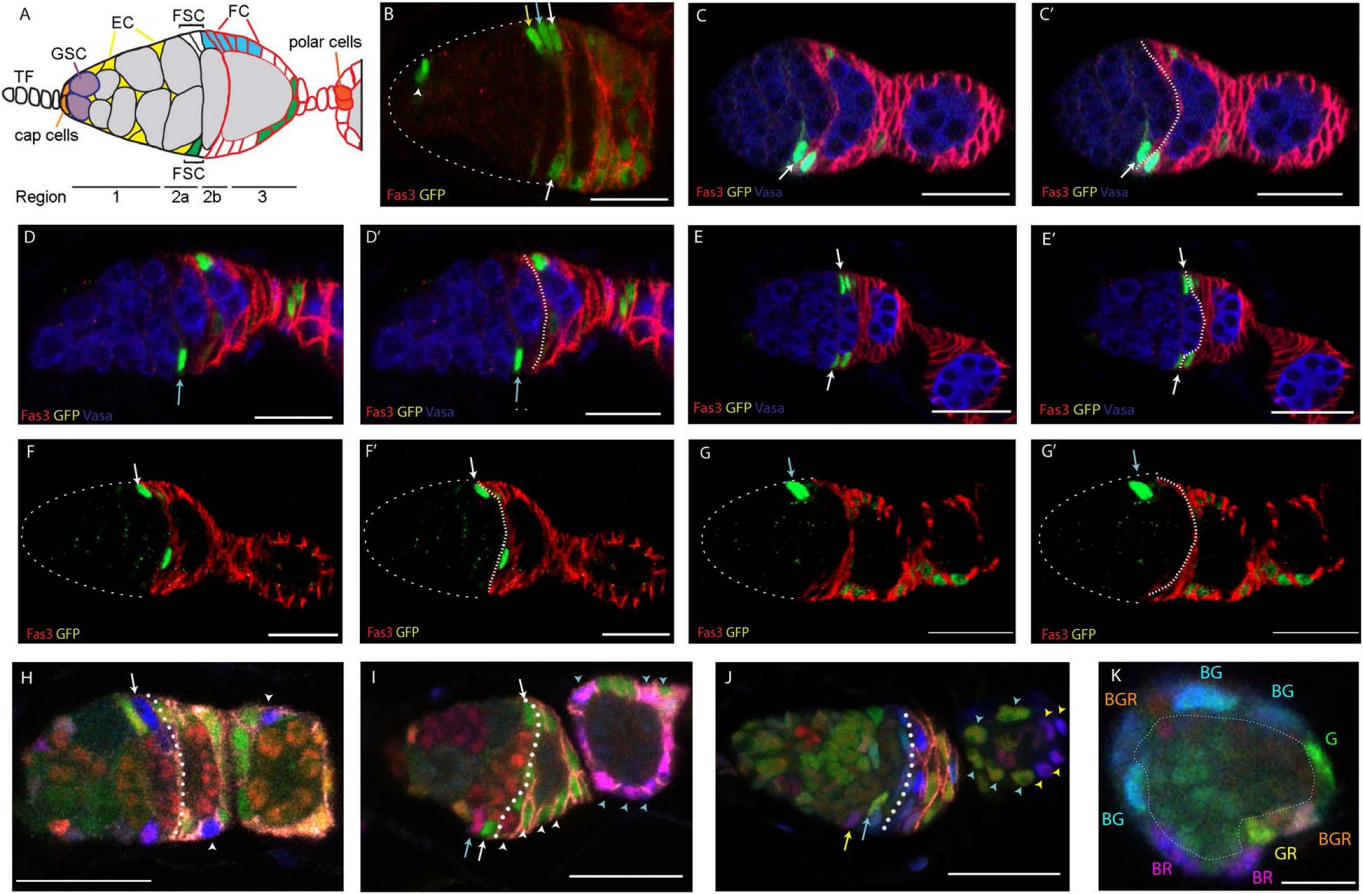
Location of FSCs in the Drosophila germarium. **A** Germline stem cell (GSC) daughters (gray) develop into 16-cell cysts as they progress further posterior (right) in region 1, supported by interactions with quiescent enveloping escort cells (ECs) and span the width of the germarium in region 2b as ECs are replaced by follicle cells (FCs). Two potential marked FSC lineages are shown (blue, green), together with the pattern of strong Fas3 surface protein expression (red). **B–J** Examples of FSC lineages marked by positive marking and multicolor lineage tracing. The anterior border of strong Fas3 generally aligns with the posterior of the stage 2b germline cyst, as in **A**, and is indicated by broken white lines in **C’**, **D’**, **E’**, **F’**, **G’**,** H–J**; central z-sections are shown but experiments examine cells in all z-sections. **B–G** show GFP-positive (green) MARCM FSC lineages with Fas3 (red) and **C–E** Vasa (blue) marking germline cells. FSC nuclei are indicated by colored arrows according to AP location of layer 1 (white), layer 2 (cyan), or layer 3 (yellow), from posterior to anterior. **B** Most FSC lineages that survive for several days contain several FSCs (arrows) and one or more labeled EC (arrowhead), precluding identification of which of these cells may be maintaining the lineage. Lineages with only a single-candidate stem cell (**C**, **D**, **F**, **G**) or with candidates in only a single AP plane (**E**) were comprehensively scored to reveal that an FSC could be found immediately anterior to the Fas3 border (white arrows), one cell further anterior (blue arrows) or occasionally one cell further anterior. **H–K** Multicolor FSC lineages, marked by the loss of GFP (**G**, green), RFP (**R**, red), or lacZ (**B**, blue) in different combinations were analyzed in the same way to reveal single candidates or single-plane candidates, as indicated by colored arrows (derivative FCs of each are indicated by arrowheads of matching color). Single-candidate FSCs were also found to be evenly distributed around the germarial circumference (in all z-sections). **K** A germarium oriented perpendicular to others shows that in cross-section around the AP plane of layer 1 FSCs there are multiple somatic cell nuclei (nine here, labeled according to retained colors) surrounding a central germline cyst. Scale bar 20 µm in **B–J**, 10 µm in **K**

At the anterior of each germarium are non-dividing somatic terminal filament (TF) and cap cells, which contact 2–3 germline stem cells (GSCs) (Fig. 1A). GSCs generally divide asymmetrically to produce a more posterior cystoblast, which divides four times with incomplete cytokinesis [19–21]. The developing germline cyst derivatives are wrapped by processes of somatic escort cells (ECs) as they progress posteriorly through region 1 [22, 23] to form a rounded 16-cell stage 2a cyst, which then elongates into a lens-shaped 2b cyst, spanning the widest part of the germarium (Fig. 1A). Proliferative follicle cell precursors (“FCs”) associate with the 2b cyst, which then rounds to become a stage 3 cyst before budding from the germarium as an egg chamber, enclosed in a monolayer epithelium of FCs. Quiescent polar cells at the anterior and posterior termini of the FC epithelium contact non-dividing stalk cells, which connect consecutive egg chambers. Polar and stalk cell precursors are specified early within the germarium [24–26], while other FCs continue to divide until mid-oogenesis (stage 6), later specializing according to their anteroposterior (AP) and dorsoventral (DV) locations on the egg chamber [27, 28]. FSCs lie between ECs and the earliest FCs (Fig. 1A), providing a continuous supply of new FCs and also, less frequently, replenishing ECs [13, 25].

FSCs, like any adult stem cells, are defined by the key functional criterion of behavior over time. Lineage assays revealed the presence of about 14–16 FSCs per germarium, distributed around the inner surface of the germarium, principally in two rings of cells (“layer 1” and “layer 2”) immediately anterior to the key landmark of the anterior border of strong surface Fasciclin 3 (Fas3) staining (Fig. 1A) [13, 17, 29]. Assigning cell locations relative to a consistent landmark is a challenging task because the three-dimensional structure of individual germaria can be irregular and a dynamic process is being sampled at random times throughout a 12 h cycle of FC recruitment. We therefore describe a detailed protocol (see “Methods”) to promote consistency for all investigators and provide numerous examples from the lineage studies summarized below (Fig. 1B–K).

The location of FSCs was inferred by examining a large number of marked FSC lineages and using those with only a single-candidate stem cell to determine FSC locations [2, 13]. This strategy indicated that FSCs were almost all either immediately anterior to the strong Fas3 border (layer 1; white arrows in Fig. 1C, E, F, H, I) or one cell further anterior (layer 2; cyan arrows in Fig. 1D, G, I, J), with a few FSCs in the next most anterior location (layer 3; yellow arrow in Fig. 1J). The total average number of layer 1 (eight) and layer 2 cells (six), distributed in radially equivalent locations abutting the germarial wall (Fig. 1K), was consistent with estimates of total FSC numbers derived from (i) measuring the average contribution of a single FSC lineage to all FCs and (ii) counting the number of different multicolored lineages in a single ovariole (in each case, taking steps to ensure capturing the full diversity of all FSC lineages, including the most short-lived) [2, 13]. After defining FSC locations in this way, it was then possible to count labeled FSCs in layers 1 and 2 explicitly in numerous studies exploring the effects of altered genotypes, regardless of the number of marked FSCs per germarium [17]. Samples in such studies typically show marked FSCs in more than one layer (surviving lineages can amplify FSCs and FSCs can move between layers), several marked FCs and a limited number of marked ECs (FSCs can become FCs or ECs) (Fig. 1B). Changes in the measured numbers and behaviors of marked FSCs due to altered genotypes yielded a self-consistent picture of FSC responses to different signaling environments [17]. Moreover, recent measurements of absolute rates of FSC division matched conversion rates to FCs and ECs [30], consistent with maintaining a stable population of about 16 FSCs. How FSCs differ from FCs and ECs, as well as potential regulators of FSC behaviors, can be informed and suggested by studying RNA profiles.

### scRNA-seq investigation of anterior somatic germarial cells

Some scRNA studies have been conducted on whole ovarioles to capture all aspects of oogenesis [18, 31, 32]. We were interested only in examining FSCs and their neighbors in detail, so we chose a strategy that began with purifying those cells. The enhancer trap line *C587-GAL4*, which is expressed in adult ECs, FSCs, and the earliest FCs, was used to drive *UAS-CD8-RFP* cell surface protein expression, followed by manual dissection of ovaries, dissociation, and fluorescence-activated cell sorting (FACS). Single-cell RNA sequences were derived on the 10X Genomics platform with unique molecular identifiers (UMIs). Average raw sequence reads per cell were over 290, 000. After genome alignment and UMI counting, genes featured in more than three cells were included, and cells with feature counts between 100 and 4500 were retained for principal component analysis using Seurat (v4.0.2).

**Fig. 2:**
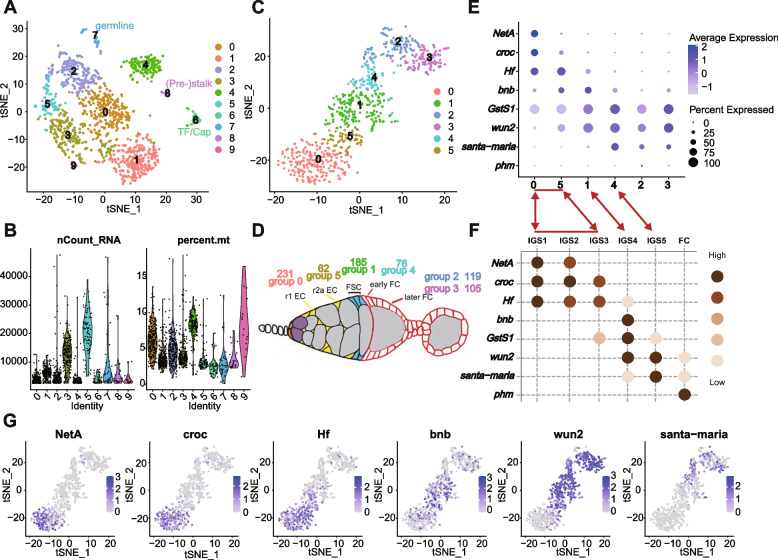
Cluster analysis of scRNA-seq profiles of germarial cells. **A** t-Distributed Stochastic Neighbor Embedding (t-SNE) plot of the dataset, together with **B** the number of unique molecular identifiers (nUMI) detected per cell and percentage of mitochondrial gene for each cluster. **C** t-SNE plot after re-clustering the original groups 1, 3, 5, and 2 only. **D** Deduced location in the germarium of cells within the six clusters colored as in **C**. **E** Summary dot plot of key marker genes defining clusters presented in **F** [40], together with dot plots of the same genes for the six clusters in **C**. Double-headed arrows indicated similar groups in the two studies (groups 0 and 5 span the content of groups IGS1-3; *phm* was not detected in our study). **G** Expression of the six most informative maker genes imposed on t-SNE plot of **C** groups 0–5. Supporting information for **C** and **E** in Additional file 2: Table 1 and Additional file 3: Table 2, respectively

Following initial clustering of scRNA patterns for a total of over 1300 cells into ten clusters (Fig. 2A), we examined each group for known indicators of cell identity. We recognized small groups with features of cap cells or TF cells (selective expression of *Lmx1a* (*LIM homeobox transcription factor 1a*), *engrailed* (*en*), *decapentaplegic* (*dpp*), and *wingless* (*wg*); 43 cells) [33–36], stalk cells and their precursors (selective expression of *Lamin C* (*LamC*; also expressed in TF and cap cells) and *single-minded* (*sim*); 23 cells) [32], and germline cells (selective expression of *zero population growth* (*zpg*), *chinmo* (*chronologically inappropriate morphogenesis*), *ovo*, *ovarian tumor* (*otu*) and *vasa*; 34 cells) (Additional file 1: Fig. S1) [32]. These three cell types do not significantly express *C587* > *CD8-RFP*, consistent with their relatively low representation (as cells escaping FACS selection). Three groups (0, 4, 9 in Fig. 2A) were not considered further on the basis of a combination of higher mitochondrial RNA content and lower total RNA counts, suggesting the possibility of damage or stress (Fig. 2B). The remaining four groups had characteristics of ECs and FSCs (*Wnt4*, *patched* (*ptc*), *failed axon connections* (*fax*)) [13, 37–39] or early FCs (*Fas3*, *castor* (*cas*)) [24] and were re-sorted by themselves in order to gain better resolution among somatic cells of the anterior half of the germarium. The result was a progression of six clusters, roughly consistent with progressive anterior to posterior identities (Fig. 2C, D; Additional file 2: Table 1 for Fig. 2C), as described further below.

**Fig. 3:**
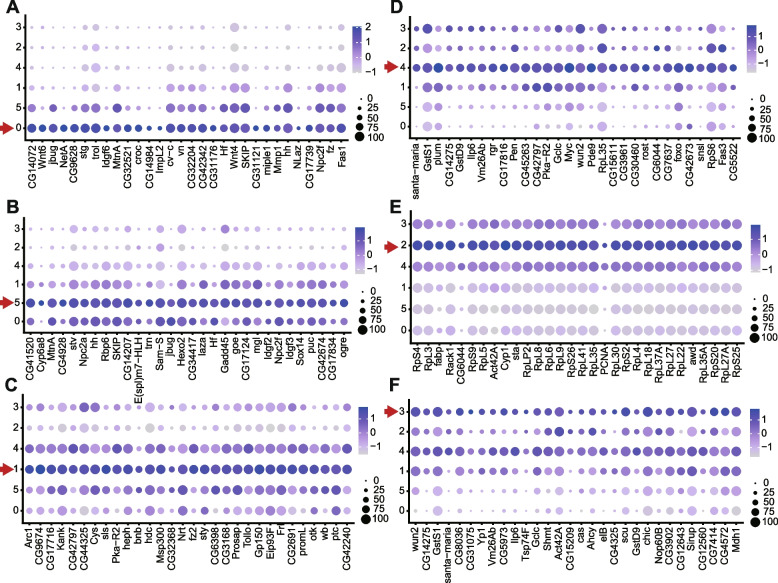
Marker genes for six clusters of somatic germarial cells. Dot plots showing the expression profiles of the top 30 markers (ordered by avg_log2FC) in each of the clusters, **A** cluster 0, **B** cluster 5, **C** cluster 1, **D** cluster 4, **E** cluster 2, and **F** cluster 3, proceeding from anterior to posterior cell groups in the germarium. Circle color intensity represents the average RNA abundance and circle size represents the proportion of cells found to express the RNA within each group

We compared our clusters to those derived from another study that also concentrated on anterior somatic germarial cells [40] and found substantial similarities. The patterns of selectively expressed RNAs that defined groups previously labeled as “IGS1-5” (IGS, or inner germarial sheath, is an alternative name for escort cells) showed largely good correspondence to our group 0 (IGS1 and 2; *Netrin A* (*NetA*) + *crocodile* (*croc*)), group 5 (IGS3; *Croc* + *Helical factor* (*Hf*)), group 1 (IGS4; *bangles and beads* (*bnb*) + *wunen 2* (*wun2*)), and group 4 (IGS5; *wun2* + *santa maria*) (Fig. 2E–G; Additional file 3: Table 2 for Fig. 2E). Importantly, Tu et al. [40] used several of the cited “marker RNAs” for in situ hybridization to define the spatial limits of specific clusters, confirming the anterior to posterior progression of groups 0-5-1-4 in that order, as described below. Clusters 2 and 3 appear to represent more mature FCs, with ribosomal protein RNAs dominating the most upregulated genes of cluster 2 (Fig. 3E), consistent with the highly proliferative nature of FCs. This feature is presaged in the earliest FCs in group 4 by strong expression of two ribosomal protein RNAs (*RpL35* and *RpS6*) and *myc* (Fig. 3D). A handful of cells within group 2 expressed JAK-STAT ligands, *unpaired 1* (*upd1*) and *unpaired 3* (*upd3*), characteristic of polar cells [41].

### Cluster boundaries and cell identities

From RNA in situs, *santa maria* is mostly expressed posterior to the strong Fas3 border, with weaker expression just anterior to the Fas3 border, overlapping with the posterior margin of *bnb* RNA [40]. *Santa maria* RNA is prevalent in group 4 but almost completely absent from group 1, while *bnb* RNA is highest in group 1 but present in some cells of group 4, suggesting that the key Fas3 border is roughly between groups 1 and 4, with most FSCs in group 1 (Fig. 2E, G). A few of the most posterior FSCs, expressing both *bnb* and *santa maria*, may be in group 4.

*bnb* expression was strongest in group 1 but also significant in abundance and prevalence in group 5 (Fig. 2E, G). Since the anterior boundary of *bnb* RNA expression in situ appears to extend beyond FSCs into region 2a (r2a) ECs [40], the transition between ECs and FSCs is likely very close to the border between groups 5 and 1. Other genes with sharp declines from group 5 to 1 include *Hf* and *CG34417* (Fig. 3B), with the converse pattern for *CG42797* and *frizzled 2* (*fz2*) (Fig. 3C). Several RNAs peak within group 1, including *headcase* (*hdc*), *Kank*, *sallimus* (*sls*), *prominin-like* (*promL*), and *sprouty* (*sty*)) (Fig. 3C).

Regarding the more anterior clusters, *NetA* RNA is restricted to within region 1, while the posterior border of *croc* RNA extends into region 2a, declining quite sharply at the anterior border of *bnb * [40]. *NetA* expression was limited to group 0, while *croc* RNA was present also in a minority of group 5 cells, suggesting that groups 0 and 5 largely represent region 1 and 2a ECs, respectively (Fig. 2E, G).

Considering only groups 0, 5, and 1 (478 cells), the total number of cells in groups 0 (231, 48%), 5 (62, 13%), and 1 (185, 39%) are roughly in proportion to the average number of region 1 (r1) ECs (26, 46%), r2a (14, 25%), and FSCs (16, 29%) in an adult germarium [13, 42, 43], suggesting rough equivalence if all cells were captured equally well for sequencing. Thus, group 1 cells appear to correspond largely to FSCs, though it is expected that a few FSCs may also be within group 4 and not all cells in group 1 are necessarily FSCs (there may be some r2a ECs and some FCs) (Fig. 2D).

### Prospective FSC labeling for lineage analyses

The previous section explained how a specific cluster was assigned FSC status based on the spatial expression profile of various RNAs relative to a landmark (the anterior border of strong Fas3 expression), which had been used to define FSC locations in functional lineage tests. The single-candidate method, described earlier, used to identify FSC locations is effective but it has not commonly been used for other stem cells. Instead, for mouse stem cells, the traditional approach has been to find a sufficiently specific marker to label a subset of cells through a recombination event and then determine by lineage analysis if such labeled cells have stem cell properties [2, 44]. In practice, there is often no single suitable marker with absolute specificity. Instead, an empirical threshold of marker gene expression is engineered to initiate a *Cre-loxP* recombination event under specific conditions of sensitivity. Consequently, questions inevitably remain about whether all cells captured by that strategy are stem cells and whether only a subset of stem cells have been captured [2]. These limitations are not commonly voiced but are significant. Despite the limitations of this targeted approach, it could usefully be applied also to FSCs, provided suitably specific targeting of cells in specific locations can be accomplished. We tried to use this approach to provide further confirmation of the location of FSCs.

**Fig. 4:**
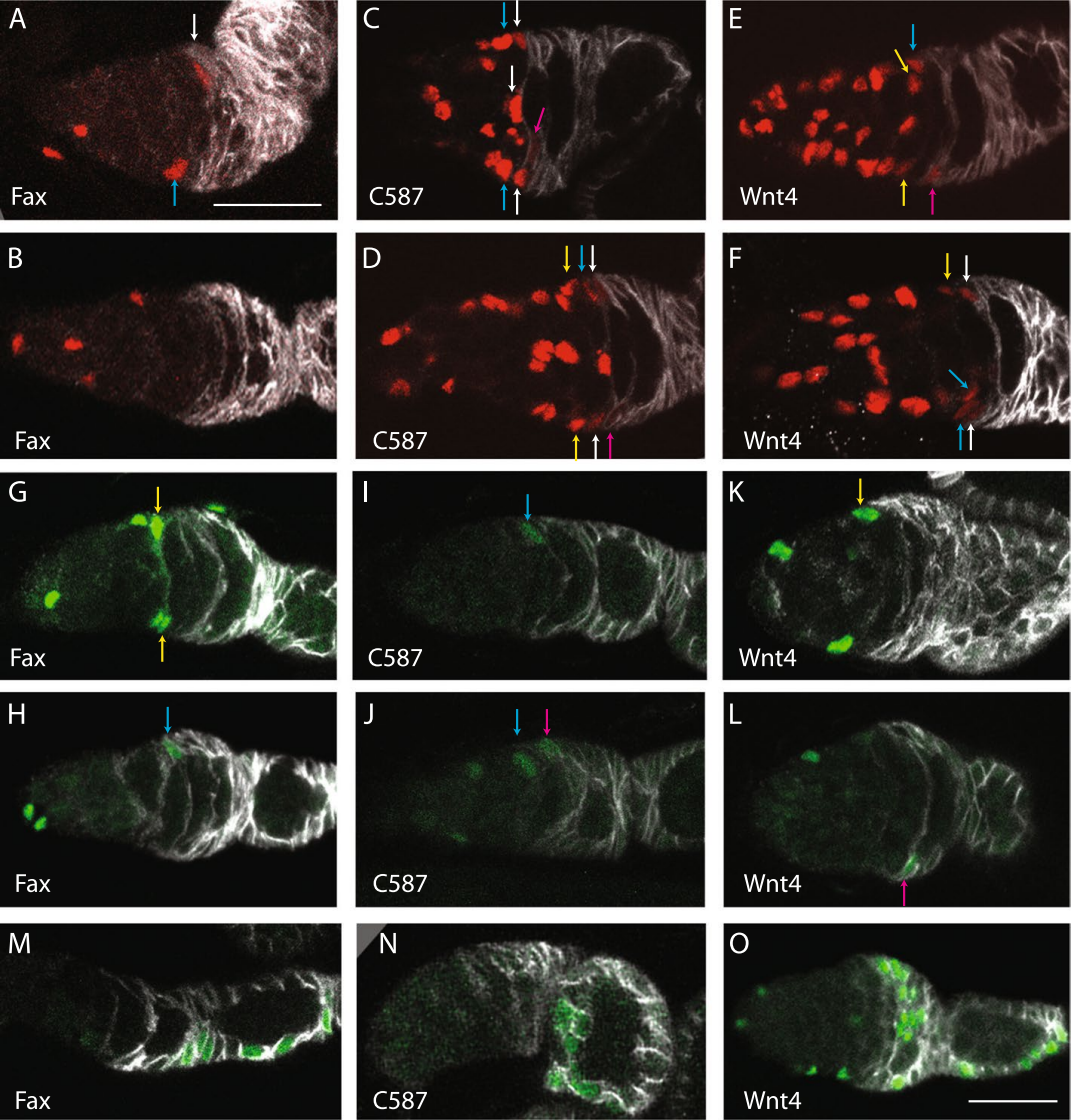
G-trace lineage analysis with *fax-GAL4*, *C587-GAL4*, and *Wnt4-GAL4*. **A–F** Expression of *UAS-RFP* after incubation at the restrictive temperature for **A**, **B**, **E**, **F** 3 days or **C**, **D** 7 days, with Fas3 antibody staining (white). In all cases, several ECs, far anterior (left) to strong Fas3 and arrows, express RFP (red). **A, B** RFP was seen in some **A** layer 1 (white arrow) and layer2 (blue arrow) cells but **B** not in all samples for *fax-GAL4*. **C, D** RFP was seen in layer 1 (white arrows), layer 2 (blue arrows), and layer 3 (yellow arrows), as well as more faintly in cells just posterior to the strong Fas3 border (pink arrows) for *C587-GAL4*. **E, F** RFP was seen in layer 1 (white arrows), layer 2 (blue arrows), and layer 3 (yellow arrows), as well as more faintly in cells just posterior to the strong Fas3 border (pink arrows) in **E** some samples but **F** not others for *Wnt4-GAL4*. **G–L** GFP (green) expression at day 1 after incubation at the restrictive temperature (30 °C) and return to 18 °C. **G, H** For *fax-GAL4*, GFP was common in ECs, occasionally present in layer 1, 2 (blue arrow), or 3 (yellow arrows) cells (24%, *n* = 108) and rarely present posterior to the strong Fas3 (white) border (6%, *n* = 108). **I, J** For *C587-GAL4*, GFP was detected in ECs, sometimes in layer 1, 2 (blue arrows), or 3 cells (24%, *n* = 58) and less often **J** immediately posterior to the strong Fas3 border (pink arrow) (14%, *n* = 58). **K, L** For Wnt4-GAL4, GFP was detected in ECs, frequently in layer 1, 2, or 3 (yellow arrow) cells (56%, *n* = 34) and almost as often **L** immediately posterior to the strong Fas3 border (pink arrow) (41%, *n* = 34). **M–O** GFP (green) expression after a further 6 days at 18 °C was found in small groups of FCs in the germarium or first three egg chambers in a subset of samples for **M** *fax-GAL4* (21%, *n* = 70), **N** *C587-GAL4* (24%, *n* = 41), and **O** *Wnt4-GAL4* (37%, *n* = 30). Scale bar of **A** applies also to **B–N**; all are 20 µm

The most common targeting strategy in Drosophila uses specific gene regulatory elements to drive yeast GAL4. GAL4 then activates a *UAS-flp* recombinase gene to initiate stable GFP expression in a recombinant cell and its derivatives (“G-trace”) [45]. Temperature-sensitive GAL80, which inhibits GAL4, is generally included, so that the timing of recombination events can be limited to a chosen time window at the restrictive temperature. The activity of a specific *GAL4* transgene does not necessarily reflect normal expression of the parent gene and is normally assayed by including a *UAS-RFP* reporter. From scRNA-seq studies, RNA in situs, enhancer trap lines and other measures of patterned gene activity, a handful of GAL4 lines have been identified as having potentially suitable spatial specificity. According to *UAS-RFP* patterns, specific *Wnt4-GAL4*, *C587-GAL4*, and *fax-GAL4* lines have been reported to be expressed largely or entirely anterior to the strong Fas3 border [46]. We found that after at least 3 days at the restrictive temperature, RFP expression was mostly anterior to the strong Fas3 border for all three lines, with strongest expression for *Wnt4-GAL4* and weakest for *fax-GAL4* (Fig. 4A–F). All *Wnt4-GAL4* germaria (24/24) included RFP-positive cells anterior to the Fas3 border in locations we have previously designated as layer 1–3 FSCs, compared to 19/27 (70%) for *fax-GAL4*. Most such cells were labeled in each *Wnt4-GAL4* germarium but only an average of 2.3 (44 total) for *fax-GAL4*. There was also detectable RFP in cells immediately posterior to the Fas3 border in about a third (8/24) of germaria for *Wnt4-GAL4* (generally one cell with weak expression). RFP label was detected posterior to the Fas3 border in very few (3/59) *fax-GAL4* germaria. For *C587-GAL4*, RFP was detected after 7 days at 30 °C in both layer 1–3 locations anterior to the Fas3 border and at least one cell posterior to the border in all cases (10/10). In the same samples, GFP patterns roughly mirrored RFP expression. However, while RFP expression provides an analog measure of GAL4 activity, GFP expression is digital, with permanent expression at a fixed level (driven by a *ubiquitin* promoter) once expression is triggered by a recombination event. Moreover, the threshold for triggering a stochastic recombination event can be quite low compared to generating a clear RFP signal. Hence, for lineage studies it is imperative to ascertain the initial GFP labeling pattern and not sufficient to rely on RFP expression patterns.

Previous G-trace tests used long periods at the restrictive temperature and reported that many ovarioles later included GFP-labeled FCs for *Wnt4-GAL4* and *C587-GAL4* [46]. Those results are broadly consistent with FSCs being located anterior to the strong Fas3 border. However, a similar experiment using *fax-GAL4* was reported to produce very few ovarioles with labeled FCs under normal conditions in well-fed flies [46]. This evidence was used to argue that FSCs lie posterior to the strong Fas3 border. We repeated those conditions for *fax-GAL4* and found substantially different results. GFP-labeled FCs were seen in 34% (17/50) of ovarioles; each of these ovarioles also included labeled cells in layer 1–3 (with an average of close to 3 labeled layer 1–3 cells per germarium), while only 9/50 included labeled cells immediately posterior to the Fas3 border. These results, in contrast to the previous report [46], are consistent with layer 1–3 cells as a source of FCs and do not appear to be consistent with the suggestion of long-lived FSCs located posterior to the AP border. Under the same conditions, *C587-GAL4* (10/10 = 100%) and *Wnt4-GAL4* (24/24 = 100% even by 3d) produced a greater frequency and density of FC labeling, but these two GAL4 lines are expressed at higher levels and in more cells anterior to the Fas3 border than *fax-GAL4*.

A much better lineage test would be to label single or very few cells in each germaria over a short time period, ensure no further labeling, and then “chase” for a period sufficient to determine if labeled FCs are formed. We experimented with a variety of conditions to achieve this outcome. A significant practical limitation here (and elsewhere [42, 47]) was the capacity of the temperature-sensitive *GAL80* transgenes to silence GAL4 activity at the permissive temperature. Even in the presence of two such transgenes, GFP clones were occasionally initiated for both *Wnt4-GAL4* and *fax-GAL4* for flies raised and aged at 18 °C. The nature of those GFP labeling patterns was variable but the frequent heavy-labeling of multiple ECs, FSCs, and FCs suggested that most recombination events occurred during development [42], when the expression patterns of *Wnt4-GAL4* and *fax-GAL4* are potentially significantly different from those in adults. *C587-GAL4* with three copies of the *ts-GAL80* transgene gave virtually no background GFP-positive clones at 18 °C. A second experimental limitation concerns timing prior to determining initial GFP-labeling patterns. For the weakest GAL4 line, *fax-GAL4*, it was necessary to incubate for 10.5 h at the restrictive temperature to have relevant cells labeled in at least 20% of ovarioles. Ideally, flies are then returned to 18 °C for long enough for all recombination events to manifest as detectable GFP signal before assaying a cohort of flies for initial (“time zero”) GFP expression patterns. However, waiting too long will allow some initially labeled cells to divide or to move to different locations, including crossing the strong Fas3 boundary. We developed near-optimal timing for each GAL4 line but the compromise between a sufficient induction period and assaying a true “time zero” was greatest for *fax-GAL4* because it had the weakest expression. Conversely, the third ideal requirement of limiting GAL4 activity and initial GFP marking *strictly* to cells anterior to the strong Fas3 border was not met by *Wnt4-GAL4* and *C587-GAL4*. An ideal single GAL4 line, with sufficiently strong and selective expression, may not be attainable because of the inevitable similarities between a layer 1 FSC and the FC that it can become within a few hours.

Using the best conditions we could establish, we conducted quantitative G-trace experiments for all three GAL4 lines. Samples were scored to ascertain initial GFP labeling patterns (day 1) and after a further 6 days at 18 °C (day 7). Since development is roughly twice as slow at 18 °C compared to 25 °C and egg chambers generally bud every 12 h at 25 °C, it is expected that the most immature FCs (“immediate FCs”) will progress out of the germarium (2 days) and occupy the 4th or 5th egg chamber after 6 days, as previously observed directly for a 3-day interval at 25 °C [9]. A FSC may simply remain as a FSC but if it or its progeny become a FC, such FCs would be in the 4th egg chamber or more anterior locations. Thus, based on our current understanding, we would expect that all marked FCs anterior to egg chamber 4 on day 7 are derivatives of initially marked FSCs (and a few FSC derivatives may also be present in the 4th egg chamber).

For *Wnt4-GAL4*, a small number of ovarioles (< 15%) at day 1 and day 7 included strong, widespread GFP labeling and presumably originated during development, as described above. These background clones were discounted and not included in totals because any additional labeling induced at the restrictive temperature in adults could not be scored. At day 1, 19/34 germaria had cells labeled in layers 1–3 anterior to the strong Fas3 border (total of 37 such labeled cells; average 1.9), and 14/34 had at least one GFP-positive cell immediately posterior to the Fas3 border (8 of these also had label in layers 1–3) (Fig. 4K, L). At day 7, the observed fraction with marked FCs anterior to the 4th egg chamber was 37% (11/30) (Fig. 4O). These results are compatible with the marked FCs at day 7 originating from either layer 1–3 cells or cells posterior to the Fas3 border. Essentially, *Wnt4-GAL4* initially labels cells posterior to the Fas3 border too frequently to provide a decisive test, even though the pattern of *UAS-RFP* labeling is strongly biased towards cells anterior to the Fas3 border.

For *C587-GAL4* at day 1, 14/58 germaria had cells labeled in layers 1–3 anterior to the strong Fas3 border (total of 20 such labeled cells; average 1.4), and 8/58 (14%) had a GFP cell immediately posterior to the Fas3 border (4 of these also had label in layers 1–3) (Fig. 4I, J). At 7 days, 10/41 (24%) of ovarioles included labeled FCs anterior to the 4th egg chamber and a further 5/41 (12%) included labeled cells in layers 1–3 with no labeled FCs (Fig. 4N). The proportion of 7-day ovarioles with labeled FCs (24%) exceeded the proportion with labeled cells posterior to Fas3 on day 1 (14%), suggesting that there must be a major source of FCs anterior to Fas3. By contrast, the proportion of germaria with labeled layer 1–3 cells at day 1 and labeled FCs at day 7 were similar, consistent with the former serving as FSCs.

For *fax-GAL4*, 15/123 ovarioles at day 1 and 11/81 ovarioles at day 7 showed heavily labeled background GFP signal and were excluded. At day 1, 26/108 (24%) of germaria had cells labeled in layers 1–3 anterior to the strong Fas3 border (total of 70 such labeled cells; average 2.7), and 7/108 (6%) had a GFP cell immediately posterior to the Fas3 border (6 of these also had label in layers 1–3) (Fig. 4G, H). At 7 days, 15/70 (21%) of ovarioles included labeled FCs anterior to the 4th egg chamber, FCs in the 4th egg chamber were the only GFP-positive cells other than ECs in 4/70 (6%) ovarioles, and a further 4/70 (6%) included labeled cells in layers 1–3 with no labeled FCs (Fig. 4M). The proportion of 7-day ovarioles with labeled FCs (24%) is much higher than the proportion with labeled cells posterior to Fas3 on day 1 (6%), showing that the major source of FCs must be anterior to Fas3. The frequency of ovarioles with labeled layer 1–3 cells at day 1 (24%) roughly matched the sum of FC derivatives (21%) and layer 1–3 cells with no FC derivative (6%) at day 7, consistent with the expected behavior of layer 1–3 cells as FSCs [2, 13]. FCs in the 4th egg chamber (6%) may have derived from FSCs or cells immediately posterior to the Fas3 border (6%). In summary, our examination of lineages targeted largely to cells anterior to the strong Fas3 expression border supports our earlier assertion that FSCs lie anterior to this border, and hence the assignment of group 1 from scRNA-seq studies as being centered on FSCs.

### Patterns of gene expression

**Fig. 5:**
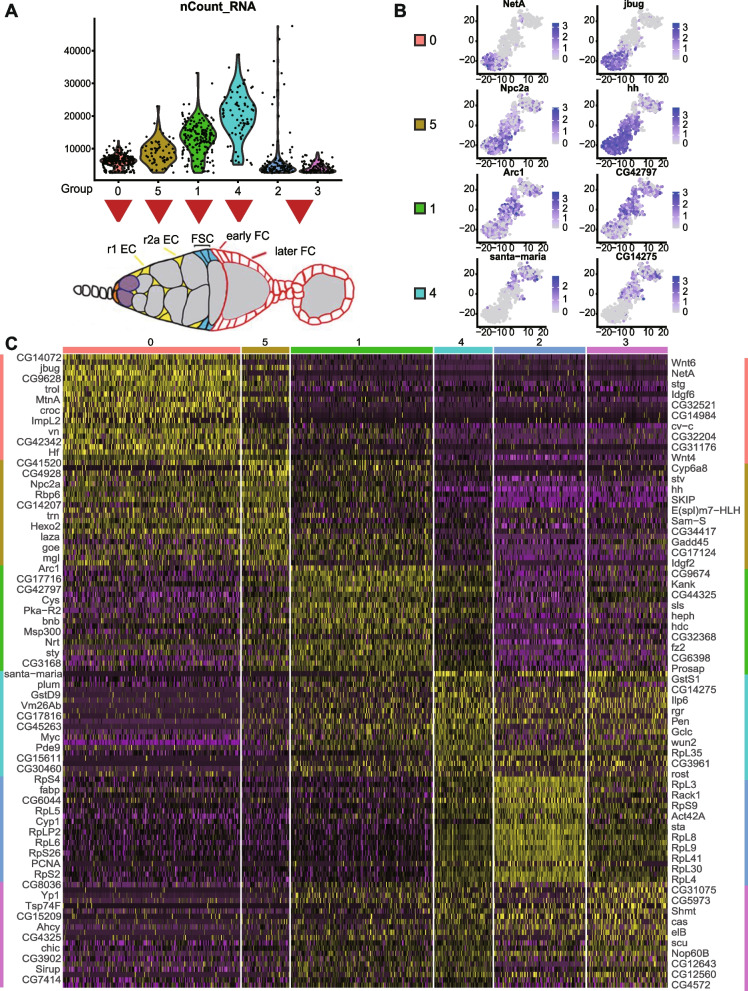
Characteristic RNA profiles for EC, FSC, and early FC groups. **A** The number of unique molecular identifiers (nCount_RNA) detected per cell is shown for each group, together with the deduced locations and cell types represented by each group. The average UMIs per group were 6203 (group 0), 8869 (group 5), 13,046 (group 1), 20,096 (group 4), 6863 (group 2), and 4072 (group 3). **B** Expression of the two maker genes in each of groups 0, 5, 1, and 4 that provide the clearest visual evidence of selective expression, imposed on the t-SNE plot of Fig. 2C groups 0–5. **C** Heat-map of the top twenty genes characteristic of each group, from ECs (groups 0 and 5), through FSCs (group 1) to early FCs (group 4) and later FCs (groups 2 and 3). Gene order was ranked by enrichment (“E”) in the appropriate group “X” according to the value of E = (log2 (1 + expression in X)/(1 + average expression in all other groups). The full set of genes with E values greater than 0.4 can be seen in Additional file 4: Table 3, and the remainder in Additional file 5: Table 4. Yellow indicates higher expression than average (black) and purple indicates lower expression, with color intensity scaled to magnitude

The patterns of gene expression emerging from scRNA-seq studies of ECs, FSCs, and FCs can provide an overview of the developmental progression among these cell types and can suggest specific genes to examine for functional roles in driving transitions from FSCs to FCs or ECs. Different displays of data are described below to suit different purposes. In all cases, the raw values of average expression of a gene (number of UMIs) per cell in each cluster were first scaled and normalized to a total of 10,000 for all 8065 genes included. Thus, each value represents the fraction of all measured RNAs in a cell representing the gene in question. This number also approximates the actual average number of counts of the corresponding RNA per cell of the cluster because the average number of UMIs per cell was 9213 (average of 1.14 over 8065 genes). The average UMI count per gene per cell was progressively higher for cells from anterior ECs (group 0; 0.8), through posterior ECs (group 5; 1.1), FSCs (group 1; 1.6) to early FCs (group 4; 2.5) (Fig. 5A). This may indicate increasing overall RNA levels but might also reflect technical differences in complete cell and RNA capture.

**Fig. 6:**
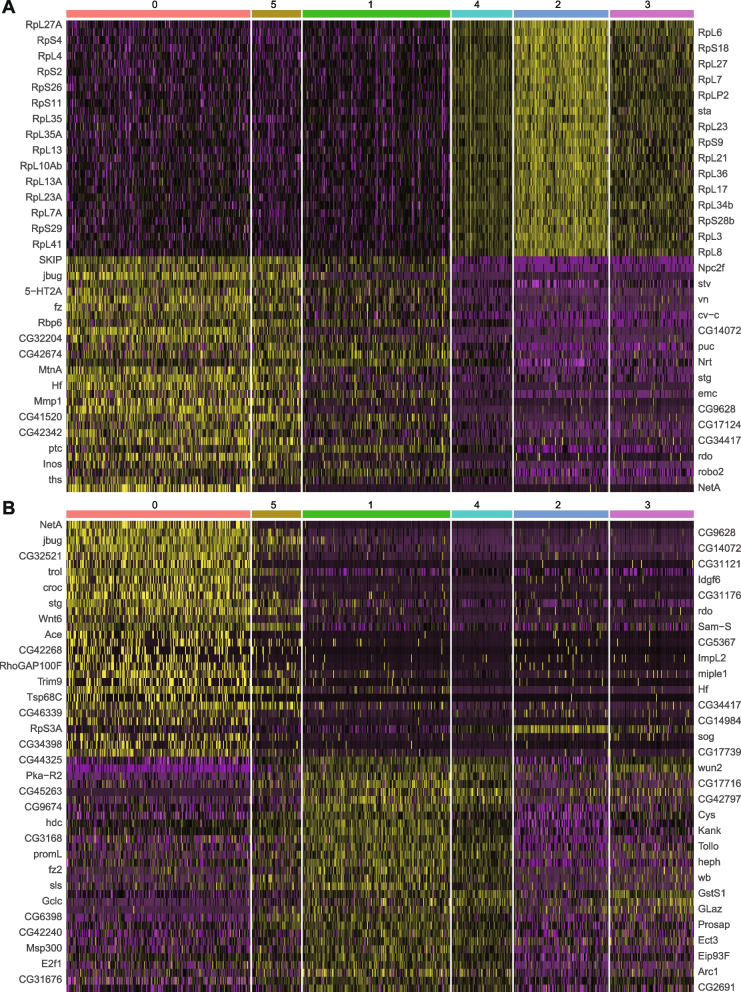
Heat-maps for the two most significant principal components. Heat-maps showing the top 30 positively correlated and negatively correlated genes for the two most significant principal components used in clustering analysis—**A** PC1 and **B** PC2. Clusters are organized from anterior to posterior (left to right), with ECs (groups 0 and 5), FSCs (group 1), and FCs of increasing maturity (groups 4, 2, and 3). The full set of genes ordered by values of PC1 or PC2 can be found in Additional file 6: Table 5 or Additional file 7: Table 6, respectively. Yellow indicates higher expression than average (black) and purple indicates lower expression, with color intensity scaled to magnitude

The normalized expression values were used first to sort genes in a manner that highlights enrichment in single clusters. Specifically, Additional file 4: Table 3 lists genes characteristic of each cluster, ranked by enrichment (“E”) in the appropriate group “X” according to the value of E = (log2 (1 + expression in X)/(1 + average expression in all other groups). Only enrichment values greater than 0.4 are listed (all values for all genes can be found in Additional file 5: Table 4), together with the percentage of cells expressing the gene within the cluster and outside the cluster, and the probability of observing this enrichment by chance. A heat-map of the top 20 genes for each cluster is presented in Fig. 5C. Heat-maps for the top 60 genes for each of the two major principal components (PC1 and PC2) used for clustering are presented in Fig. 6. Looking at all three heat-maps, the most prominent, or sharpest, transition in expression is between groups 1 (FSCs) and 4 (early FCs). This pattern is especially obvious for PC1 (Fig. 6A; Additional file 6: Table 5) but also apparent principally for genes characteristic of group 5 and group 2 (Fig. 5C), suggesting that many of the relevant genes are characteristic of EC and FC function, respectively. The genes that are activated in group 2 (FCs) and PC1 are dominated by ribosomal protein genes, and also include genes encoding products involved in ribosome assembly (*stubarista*; *sta*) and DNA replication (*Proliferating Cell Nuclear Antigen*; *PCNA*). This is consistent with the designation of groups 4 and 2 as highly proliferative FCs, groups 0 and 5 as non-proliferating ECs, and group 1 as FSCs with variable and relatively low proliferation rates.

The second-most striking transition, highlighted by PC2 (Fig. 6B; Additional file 7: Table 6) and evident for genes characteristic of groups 0, 5, 1, and 4, is between groups 5 (posterior ECs) and 1 (FSCs) (Fig. 5C). Genes that are highly expressed in ECs (group 5 and group 0) and reduced in group 1 (FSCs) are generally further reduced in FCs of groups 4 and 2. However, expression of some of the genes that are largely repressed in ECs and activated in FSCs (group 1) decline in group 4 and even more prominently in group 2 FCs, suggesting that some aspects of the EC to FSC transition are dissimilar to those of FSC to FC transitions. These patterns, emphasized by PC1 and PC2, align with previous in situ patterns of gene expression assayed by reporters and antibody studies, showing several genes expressed in ECs, declining in FSCs and terminating close to the first FCs (including the Wnt pathway activity reporter, Fz3-RFP, as well as the *PZ1444-lacZ* and *C587-GAL4* enhancer traps), but none with a sharp transition between ECs and FSCs [13, 32, 40]. These same characteristics are also evident when examining the same data as displayed in the heat-maps as “dot plots”, which additionally display visually the percentage of cells with detected transcription of each gene (Fig. 3). Several genes characteristic of group 4 or 2 (FCs) have comparatively low expression in group 1 (FSCs) (Fig. 3D, E), whereas most genes characteristic of groups 5 and 0 (ECs) do not decline greatly in FSCs (group 1). A couple of exceptions, which may be interesting to pursue functionally, are *jitterbug* (*jbug*), encoding a Filamin-type protein [48] and *Helical factor* (Hf), encoding a secreted cytokine. This pattern of *Hf* expression was noted and shown by RNA in situs in a prior scRNA-seq study [40]. Whether examined by heat-maps (Fig. 5C and Fig. 6), t-SNE plots (Fig. 5B), or dot plots (Fig. 3), strictly FSC-specific (group 1) expression is not evident. Prominently expressed genes are also expressed significantly either in ECs or FCs.

**Fig. 7:**
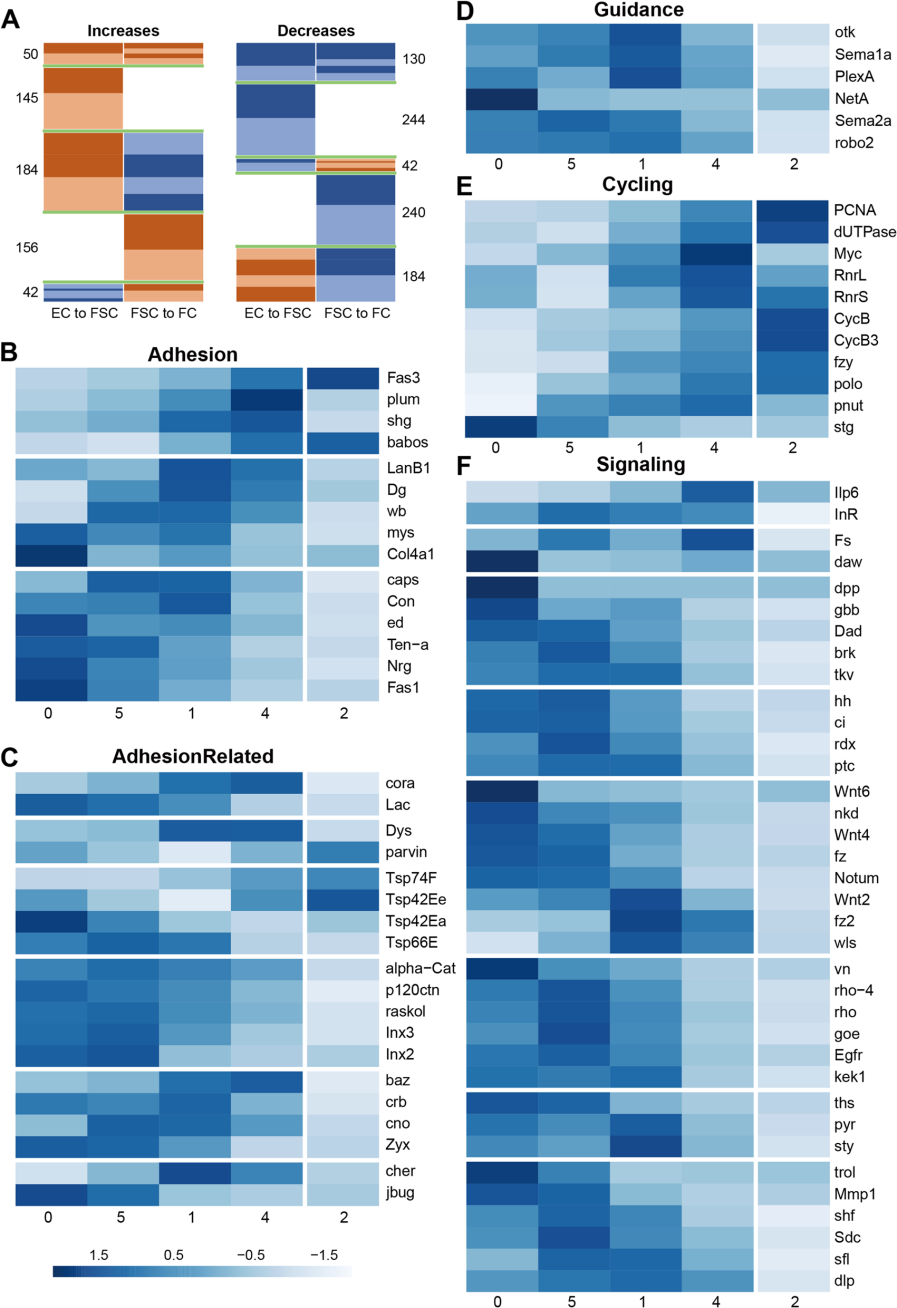
Potential regulators of cell behavior with AP-graded expression patterns. **A** Diagram of changes in gene expression for transitions from ECs to FSCs (first column) and FSCs to FCs (second column) for genes with the largest differences. Genes in the left pair of columns are displayed in the same order in Additional file 8: Table 7, while those on the right are in Additional file 9: Table 8. Genes in the first two quartiles are colored in brown for increases, and in blue for decreases, with darker colors representing the first quartile (no color indicates the 3rd or 4.th quartile, whether there are increases or decreases). Green lines are added to distinguish the different categories, with the number of genes in each category listed to the side. For example, 50 genes for UP-UP, 145 genes for UP-NEUTRAL, 184 genes for UP-DOWN, and so forth. Heat-maps of selected genes for the categories of **B** adhesion molecules, **C** adhesion-related molecules, **D** axon guidance molecules, **E** drivers or regulators of cell cycling, and **F** selected signaling molecules with pronounced differences in expression from ECs (groups 0 and 5), through FSCs (group 1) to early FCs (group 4), showing also expression in later FCs (group 2). Blue color intensity indicates relative expression level, as indicated by the logarithmic scale bar

In an adult ovary, the key regulated behaviors of FSCs are division and differentiation. Differentiation at the anterior face of the FSC domain is to ECs, while posterior FSCs directly give rise to FCs. Both differentiation events are influenced by the strength of Wnt signaling and JAK-STAT signaling [13, 17, 38], but the mediators of these two differentiation events are unknown and may be quite different. Wnt pathway activity declines from anterior to posterior over the FSC domain, while the JAK-STAT signaling gradient has the opposite polarity [13, 17, 38, 49]. Some mediators of these signals might therefore be expected to show graded expression from ECs through FSCs to FCs, with either polarity. However, this straightforward correspondence is likely often modified because some mediators may be dedicated to regulating only one transition of FSCs and patterns of gene expression of potential mediators are likely influenced by additional signals. It is therefore of interest to display scRNA-seq data in a manner that highlights genes with significantly graded expression between ECs and FSCs, between FSCs and FCs, or both. Some of these genes may be responsive to Wnt or JAK-STAT signals and may directly regulate division rate or differentiation to ECs or FCs. The spreadsheets in Additional files 8 and 9 report fractional increases or decreases, always relative to the larger number, for transitions from ECs to FSCs and from FSCs to FCs. Only genes with an average expression value per cell greater than 1.5 in at least one group were included to focus on the most robustly supported changes. Genes are ranked according to the magnitude of these differences (increases from anterior to posterior in Additional file 8: Table 7, decreases in Additional file 9: Table 8, opposing changes between the two transitions in both spreadsheets), featuring only the top two quartiles (color-coded) for each transition (values for all genes can be found in Additional file 5: Table 4). Gene order within each set was determined by the magnitude of fractional changes after combining groups 0 and 5 for ECs, and combining groups 4 and 2 for FCs. The same calculations are reported and color-coded for transitions from groups 5 to 1 and from groups 1 to 4. The color-coded patterns for EC to FSC and FSC to FC transitions are reproduced in Fig. 7A. Since there are two top quartiles for increases (“UP”) and decreases (“DOWN”), and four lower quartiles for increases together with decreases (“NEUTRAL”), random association of changes from EC to FSCs and FSCs to FCs would result in 25% of UP for one transition being associated with UP or DOWN for the second transition (50% associated with NEUTRAL). There are some major departures from this pattern. Naming changes in the EC to FSC transition first, the frequencies of UP-UP (50/379; 13%) and DOWN-UP (42/416; 10%) are very low, while the frequency of UP-DOWN is very high (184/379; 49%) compared to the default expectation of 25% frequency. The much higher frequency of UP-DOWN (184/379; 49%) compared to UP-UP (13%) indicates that the changes between EC and FSC states differ substantially from those during a FSC to FC transition.

Some of the genes that are regulated principally by Wnt or JAK-STAT signaling and involved in promoting the FSC to FC transition might be expected to fall in the UP-UP category. Within this relatively small category (50 genes) are ten genes involved in ribosome biogenesis (rRNA processing: *Nop60B* (*Nucleolar protein at 60B*), *Fib* (*Fibrillarin*), *Nop56*; 60S biogenesis: *Non1* (*Novel nucleolar protein*); ribosomal proteins: *RpL35*, *RpL5*, *RpS19a*) or protein translation (translation initiation factors *eIF1b*, *eIF1d*, *eIF3a*), another two linked to DNA replication and proliferation (*PCNA*, *dUTPase* (*deoxyuridine triphosphatase*)), a Myc collaborator (*modulo; mod)*, genes related to nuclear transport (*Pendulin; Pen*, *Ran*) and to fatty acid oxidation (*Enoyl-CoA hydratase, short chain 1*; *Echs1*). Of the three transcription factors in this group (*cut*; *ct*, *castor*; *cas*, *hairy*; *h*), Cut contributes to FC proliferation [50] and Castor is a key marker of early FCs, which later collaborates with eyes absent (Eya) to specify different types of FC [24]. Finally, Fas3, like Cas, is a key marker that increases from FSCs to FCs, while insulin-like peptide (Ilp6) is potentially important to stimulate insulin/PI3-kinase pathway activity, which is known to affect FSC division rates [51].

### Potential regulators of FSC behavior

To identify further potential regulators of FSC transitions, we prioritized the nature of the gene rather than the absolute hierarchy with regard to magnitude of changes, or comparison of changes from EC to FSCs versus FSCs to FCs. The process of differentiation of FSCs to ECs or FCs is not well understood but it certainly involves cell movement along the AP axis. Adhesion proteins are therefore likely important mediators, potentially promoting posterior FSCs joining a nascent FC epithelium, anterior FSCs moving towards ECs, or FSCs moving along a gradient of extracellular matrix (ECM) adhesion ligands. We therefore looked for adhesion molecules (and some partners and regulators) with a clear gradient of expression from group 5 through group 1 to group 4 (Fig. 7B, C). Among cell–cell adhesion molecules with increasing expression from ECs (group 5) to FCs (group 4) were *Fas3*, *DE-cadherin* (*shg*), and immunoglobulin superfamily genes, *Plum* and *babos*. The opposite pattern was identified for *Fasciclin I* (*Fas1*), *echinoid* (*ed*), *Neuroglian* (*Nrg*), *Tenascin accessory* (*Ten-a*), *connectin* (*con*), and *capricious* (*caps*). Among these genes, only the role of DE-cadherin in FSCs has so far been investigated [52]. An increase in expression from posterior ECs (group 5) to FSCs (group 1), followed by a decline in FCs (group 4) was seen for three ECM components (*Collagen type IVa1* (*Col4a1*) and the Laminin subunits, *wing blister* (*wb*) and *LanB1*), an integrin β subunit (*myospheroid*, *mys*), and the major non-integrin ECM receptor (*Dystroglycan* (*Dg*).

A subset of genes encoding proteins involved in organizing adhesion complexes or connecting them to the actin cytoskeleton is illustrated as “adhesion-related” (Fig. 7C), and grouped according to prior evidence of involvement in septate junctions (*coracle* (*cora*) and *Lachesin* (*Lac*)), ECM interactions (*Dystrophin* (*Dys*) and *parvin*), microdomain-clustering Tetraspanins (*Tsp74F*, *Tsp42Ee*, *Tsp42Ea*, *Tsp66E*), Cadherin collaborators (*a-catenin* (*a-cat*), *Adherens junction protein p120* (*p120ctn*), *raskol* GTPase and Innexins, *Inx 2* and *Inx3*), other adherens junction proteins (*bazooka* (*baz*), *crumbs* (*crb*), *canoe* (*cno*) and *Zyxin* (*Zyx*)), and Filamin-family actin cross-linkers *cheerio* (*cher*) and *jitterbug* (*jbug*). Genes within most of these categories show opposing patterns of expression but Cadherin collaborators all decreased from ECs through FSCs to FCs, in contrast to DE-cadherin (*shg*) itself.

Several of the adhesion molecules described above are used for axon guidance (including Fas3 and Fas1, Nrg, Caps, and Con). Significant changes in gene expression were also found for guidance molecules not directly involved in cell adhesion (Fig. 7D), including a Netrin ligand (*NetA*), two Semaphorin ligands (*Sema1a*, *Sema2a*), two receptors for a repulsive Sema 1a signal (*off-track* (*otk*) and *PlexinA* (*PlexA*)), and a receptor for Slit ligand (*roundabout 2* (*robo2*)).

Posterior FSCs cycle faster than anterior FSCs, while ECs do not divide at all [13]. Graded JAK-STAT activity, declining from the posterior, is one contributor to this patterned proliferation, while Integrin, Hedgehog (Hh), and Phospho-inositide 3’ kinase (PI3K) pathways also provide major stimuli for FSC division [17, 51, 53–55]. Most ECs are found in G1, while most posterior FSCs are in G2, indicating limitation of cycling by G2/M restriction in all FSCs with an increasingly severe G1/S restriction further anterior [30]. We found that several RNAs associated with DNA replication increased from anterior (groups 0 and 5) to posterior (group 4), including *Proliferating Cell Nuclear Antigen* (*PCNA*), *deoxyuridine triphosphatase* (*dUTPase*), *Ribonucleotide reductase small* (*RnrS*), and *large* (*RnrL*) subunit genes. A similar, graded increase in drivers of mitosis was seen, including *fizzy* (*fzy*), *polo*, *cyclin B* (*cycB*), and *cyclin B3* (*cycB3*), though *string cdc25 phosphatase* (*stg*) had strongly graded expression of opposite polarity (Fig. 7E).

Signaling pathway activities can be indicated by the magnitude of expression of ligands, although the range of ligand movement can be variable and regulated. Less frequently, pathway activity is dependent on the expression level of receptors, which are also sometimes upregulated or downregulated in response to signal. Additionally, some genes are known to respond transcriptionally to pathway activity, often as part of a negative feedback circuit, but such expression is sometimes tissue-specific, rather than universal. These gene categories that were found to have significantly patterned expression are grouped according to the signaling pathway in Fig. 7F. *Insulin-like peptide 6* (*Ilp6*) expression increased prominently towards the posterior, with *insulin receptor* (*InsR*) expression fairly uniform over the critical group 5-1-4 domain. The activin-like agonist *dawdle* (*daw*) was notably high in group 0 ECs, while expression of the activin antagonist *Follistatin* (*Fs*) [56] increased either side of the FSC region. BMP ligands (*decapentaplegic* (*dpp*), *glass bottom boat* (*gbb*)), two genes commonly induced by pathway activity (*Dads against dpp* (*Dad*) and *brinker* (*brk*)) [57], and the receptor *thickveins* (*tkv*), all decreased towards the posterior. BMP signals supplied to GSCs are strictly limited to the anterior of the germarium [58]. *Hedgehog* (*hh)* ligand, the pathway’s transcriptional effector (*cubitus interruptus* (*ci*)) and two genes generally induced by the Hh pathway, *roadkill* (*rdx*) and the Hh receptor *patched* (*ptc*) [59], were also all reduced towards the posterior. This is consistent with prior detection of *hh* expression in anterior germarial cells, supplementing very strong expression in cap cells and terminal filament cells, and a slow decline of pathway reporter activity in the posterior half of the germarium [60, 61]. A number of Wnt ligands (*Wnt6*, *Wnt4*), one receptor (*frizzled*, *fz*), and two genes often induced by Wnt-β -catenin pathway activity (*naked* (*nkd*) and *Notum*) [62, 63] also declined from anterior to posterior over the whole domain extending from ECs to FCs, while another ligand (*Wnt2*), receptor (*frizzled 2* (*fz2*)), and facilitator of Wnt secretion (*wntless* (*wls*)) only declined prominently from FSCs (group 1) to FCs (group 4). *Wnt6* has previously been found to be expressed only in anterior ECs, while *Wnt4* is strongly expressed in all ECs, tapering off at the FSC border [39, 43, 61]. The RNA expression pattern profiles described are consistent with the observed anterior to posterior decline of a Wnt pathway reporter over the FSC domain [13, 49]. *Epidermal growth factor receptor* (*EGFR*), one of its ligands *vein* (*vn*), *rhomboid-4* (*rho-4*), and *rhomboid* (*rho*), which process EGFR ligands including Spitz, all decline from group 5 through group 2 (Fig. 7F). However, the same pattern is seen for *gone early* (*goe)* which can attenuate EGFR signaling [64], and a gene often induced by EGFR pathway activity, *kekkon1* (*kek1*) [65], does not decline from ECs to FSCs. Two fibroblast growth factor (FGF) ligands, *pyramus* (*pyr*) and *thisbe* (*ths*), decline from group 5 to group 1, while the FGF antagonist *sprouty* (*sty*) undergoes the converse change. The majority of genes involved in stabilization and spread of multiple ligands through protease activity (*Matrix metalloprotease 1* (*Mmp1*)), as a glypican (*dally-like protein* (*dlp*), as heparin sulfate proteoglycan (HSPG) proteins (*Syndecan* (*Sdc*) and Perlecan, *terribly reduced optic lobes* (*trol*), HSPG associated proteins (*shifted* (*shf*) or contributing to their biosynthesis (*sulfateless* (*sfl*)) decreased consistently from anterior (ECs) to posterior (FCs). Among these, *Mmp1*, *dlp*, and *sfl* have specifically been associated with Wnt signaling [49, 66], *dlp* and *shf* with Hh signaling [67, 68]. Altogether, the expression patterns of several genes derived from these scRNA-seq studies are in accord with existing evidence of an anterior to posterior decline of some signaling pathways (BMP, Hh, Wnt) and provide some new indications of regionalized activity for others (activin, EGF, FGF), which may motivate further functional investigation.

### Independent assessment of graded expression patterns suggested by scRNA-seq

We used available reagents for immunohistochemical examination of selected gene activities highlighted by our scRNA-seq studies as potential spatially regulated mediators of FSC behavior.

**Fig. 8:**
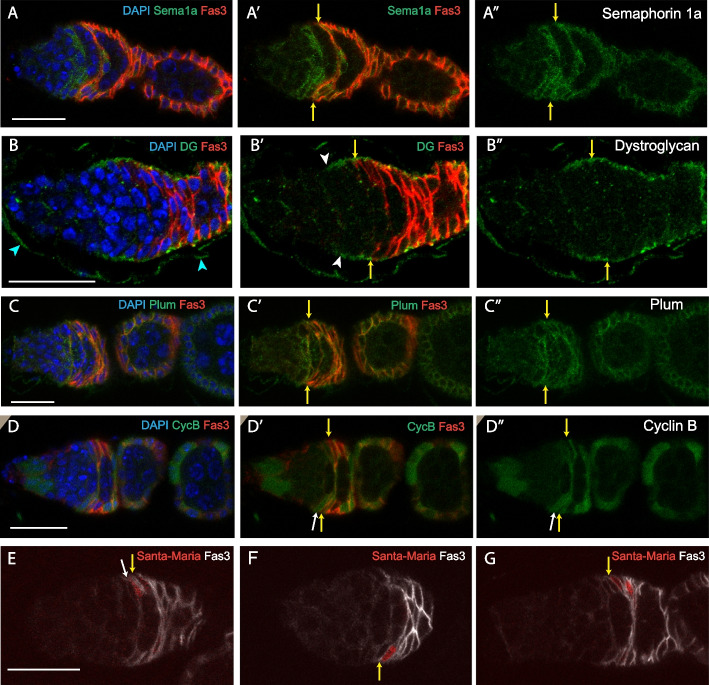
Expression patterns of selected genes. **A–F** Expression patterns of named gene products or derivatives in germaria co-stained with DAPI (blue in **A–D**) to reveal all nuclei and antibody to Fas3 (red in **A–D**; white in **E–G**) to allow location of the anterior edge of strong Fas3 expression (yellow arrows). **A–A”** Semaphorin 1a-GFP protein fusion (green) was detected on the surface of somatic cells; levels were low in region 1 (extreme anterior, left), peaked in region 2a ECs, FSCs, and early FCs, and declined in region 3 (posterior of the germarium) and the first budded egg chamber. **B–B”** Dystroglycan, detected by an antibody to the C-terminus (green), which connects the plasma membrane to ECM components, was seen lining the germarial wall from region 2a (white arrowheads in **B’**) and continuing posterior, consistent with its expected role and expression in all FSCs, early FCs, and a limited number of the most posterior ECs. Expression was also seen in the muscle sheath (cyan arrowheads in **B**). **C–C”** A Plum-GFP protein fusion (green) encoded by a large BAC genomic transgene was expressed most prominently in somatic cells, with highest levels in the FSC region and further posterior. **D–D”** A Cyclin B-GFP protein fusion showed occasional expression in layer1 FSCs (white arrow in **D’** and **D”**) immediately anterior to the Fas3 border and was seen at similar levels in most FCs. Absence in some FCs is most likely due to protein degradation in G1 and S phases. Expression at the anterior of the germarium is within germline cells. **E–G** *Santa Maria-Gal4* > *UAS-RFP* produced no RFP signal in most germaria. In other germaria, few cells expressed detectable RFP, including germaria with RFP signal in **E** a layer 1 FSC (white arrow) and a neighboring FC, **F, G** an FC adjacent to the Fas3 border, and **G** another early FC anterior to region 3. All bars, 20 µm

Semaphorin 1a (Sema1a) is a transmembrane protein that can bind to PlexA receptors on adjacent cells to counter cell adhesion in the context of axon guidance [69]. However, Semaphorin family proteins have been found to affect the migration of several other cell types, including Drosophila FCs [70]. A protein trap insertion showed expression initiating close to the EC/FSC interface and continuing into early FCs and then declining, decorating the long membrane extensions of FSCs and early FCs (Fig. 8A). This pattern strongly resembles that inferred from scRNA-seq (Fig. 7), which also predicts a largely overlapping pattern for PlexA. It will be interesting to compare Sema1a and PlexA protein distributions in the same samples to indicate whether their interactions might contribute to segregation of specific pairs of cells.

Dystroglyan, along with integrin, is a major receptor for ECM proteins and is typically found on the basal surface of epithelial cells [71, 72]. We found Dystroglycan protein to line the surface of the germarium, adjacent to the basement membrane in the most posteriorly located ECs, all FSCs, and early FCs (Fig. 8B). This pattern was very similar to the pattern of cells expressing Sema1a-GFP and the pattern inferred from scRNA-seq (Fig. 7). The pattern of Dystroglycan expression suggests it may contribute to restricting FSC to EC conversions through ECM adhesion.

Plum is an immunoglobulin superfamily protein, first functionally identified from involvement in axon pruning [73]. Plum can promote homophilic cell adhesion but may instead act by modulating TGF-β signaling in mushroom body neurons [73]. A Plum-GFP protein fusion encoded by a genomic transgene was expressed only at low levels in the anterior of the germarium but increased on the surface of FSCs and early FCs (Fig. 8C). The increase in Plum-GFP was further posterior than for Sema1a-GFP and Dystroglycan, consistent with the comparative scRNA-seq profiles (Fig. 7), but did not reflect a further increase from FSCs to FCs as suggested by scRNA-seq results. Hence, differential Plum expression is perhaps more likely to affect FSC to EC rather than FSC to FC transitions.

Recent examination of FSCs expressing fly-FUCCI reporters of cell cycle phase indicate that both G1/S and G2/M transitions can be rate limiting, with a prominent gradient of greater G1/S flux from anterior ECs through posterior FSCs [30]. Our scRNA-seq profiles suggest that a similar profile of CycB levels, promoting the G2/M transition, may also be relevant to the faster cycling of posterior FSCs than anterior FSCs. A CycB-GFP protein trap showed no significant expression in ECs and even anterior FSCs but sharply elevated expression more posterior, seen occasionally in posterior (Layer 1) FSCs and in most early FCs (Fig. 8D). Protein degradation during G1 and S phases may account for some of the observed pattern [74], but the majority of FSCs are in G2, so the absence of CycB-GFP in anterior FSCs is consistent with a deficit in the corresponding RNA. CycB, as suggested by scRNA-seq data, is therefore a prime candidate as a spatially mediator regulator that may limit FSC cycling.

The expression of *santa maria* has been noted in our scRNA-seq studies and others [32, 40, 46] to be highly selective within the germarium and therefore an important potential indicator of cell type. In our work, *santa maria* is characteristic of group 4, the earliest FCs, with significantly higher expression than in their immediate neighbors, FSCs (and also, more mature FCs). We examined the expression of *santa maria* by using a transgene with GAL4 linked to a 2 kb *santa maria* gene promoter to drive *UAS-RFP* [75]. The conditions were analogous to those described earlier for other GAL4 lines (29 °C for 7 days in the presence of temperature-sensitive GAL80). We found that germaria generally had few or no RFP-labeled cells (more than 80%), consistent with relatively low-level expression of this gene overall and stochastic variations in cells of equivalent positions and natures. RFP was seen most often in “immediate FCs”- those FCs that first have elevated Fas3 protein staining and were most recently produced (14 cells among 11germaria) (Fig. 8E–G). There were also a few additional, more mature neighboring FCs marked (11 cells) (Fig. 8G) and a small number of marked posterior FSCs (7 cells in total) (Fig. 8E). This evidence is consistent with the scRNA-seq data and group assignments. It also confirms that some FSCs may be captured among primarily early FCs in group 4. While such overlaps can reflect technical limitations of scRNA-seq, the variable *santa maria* > *RFP* expression patterns expose a fundamental issue; there is certain to be considerable overlap in gene expression between two cell types that are physically adjacent and regularly interconverted within a few hours under normal physiological conditions.

## Discussion

Single-cell RNA sequencing has great potential for understanding key regulators of a cell state. A first step in realizing this potential is to relate single-cell profiles to cell identities. For FSCs, the key intermediate is spatial location. Here, we used cells pre-sorted to represent mainly anterior germarial cells (ECs, FSCs, and early FCs) and standard bioinformatic analyses to derive clusters guided by similarities. The clusters were notably similar to those of another study [40], facilitating assignment of group locations according to prior RNA in situ results for key indicators of those groups. The cluster profiles themselves showed a clear progression consistent with assignments along the AP axis and the placement of a key spatial landmark, the anterior border of strong surface Fas3 protein expression, roughly at the border between two groups (“1” and “4”). The anterior limit of group “1” was placed about three cells further anterior by comparison to RNA in situs, forming a cluster size of roughly the expected size to represent FSCs relative to the more anterior groups (“0” and “5”) representing ECs. Importantly, this study is the first to assign the FSC group by taking into account the most comprehensive functional investigation of FSC numbers and locations [13]. Some other studies sampled the whole ovariole and therefore lacked sufficient FSCs in their samples [31, 32, 46]; others did not acknowledge recent developments in understanding FSC biology or simply avoided an FSC designation, focusing instead on other cell types [40, 46, 47]. Here, we included images of a variety of GFP-marked FSC lineages to illustrate FSC locations in 2 or 3 AP layers anterior to the Fas3 border, as determined previously by examining lineages with only a single-candidate FSC [13]. We also included a protocol for identifying the Fas3 border because other investigators have presented inconsistent and potentially confusing approaches and summaries [46].

We also undertook a complementary approach to verify FSC locations through prospective “G-trace” labeling [45]. We obtained results consistent with our prior designation of FSCs occupying 2 or 3 layers anterior to the Fas3 border and contradicting an assertion that FSCs are posterior to the AP border [46], based principally on using the very same *fax-GAL4* reagent to initiate clones. Crucially, our tests involved carefully controlled conditions that allowed production and measurement of a suitable number of GFP-labeled cells during a short labeling period, followed by a chase period to ascertain the fate of such cells. Results with *fax-GAL4* and *C587-GAL4* to initiate labeling were consistent with FC-producing cells lying anterior to the Fas3 border, although that conclusion was less numerically robust for *C587-GAL4* because the associated initial GFP-labeling pattern was less specific than for *fax-GAL4*. The principal limitation of G-trace in this context was the failure of each *GAL4* line to show absolute spatial specificity. A second limitation was that the shortest practical time between initiation of targeting recombination and assessing the cellular specificity of labeling is sufficiently long that some stem cells will differentiate and change location in the interim. These limitations to targeted labeling likely apply to most stem cell paradigms, especially those governed by population asymmetry. The best method for determining FSC locations therefore remains the single-candidate method, which allows single-cell resolution of FSC positions [2, 13]. The deduced locations and numbers of FSCs fit extremely well with many measurements of FSC division rate and FC production [2, 17, 30], as well as the targeted G-trace studies presented here.

The assignment of a group of scRNA expression profiles to FSCs is not precise for a number of reasons, including imperfect and limited depth of RNA profiles, latitude in spatial correspondence according to RNA in situs, and the systematic limitation that FSCs become ECs or FCs within a few hours and therefore inevitably have strong similarities to those neighboring derivatives. Nevertheless, the results of this study have the unique virtue that the approximately defined FSC group of cells is in accord with current understanding of FSC numbers and locations determined by thorough functional analyses [2, 9, 13, 17, 76–78]. FSC behavior is guided by a number of external signals but with prominent roles for two graded signaling pathways, Wnt, and JAK-STAT [13, 17]. RNAs that also have graded AP expression from ECs through FSCs to early FCs are therefore of special interest as candidate mediators of spatially appropriate behavior guided by these graded signals. We have highlighted such RNAs encoding potential effectors of cell movements and differentiation or cell division as candidates for further investigation of functional roles and potential transcriptional regulation by Wnt or JAK-STAT pathways.

## Conclusions

We have sorted cells from the Drosophila germarium, performed single-cell RNA sequencing and grouped clusters of profiles according to similarity. Comparison of selectively expressed RNAs to previously determined RNA in situ patterns showed a clear AP progression among four identified clusters, including the identification of one cluster as overlapping FSC territory. We confirm previous allocation of FSC locations along the AP axis using lineage analyses. Specific candidate genes with graded expression around the FSC domain are highlighted as potential effectors of FSC division and differentiation instructed by graded extracellular signals. Although other recent scRNA-seq studies have examined cells in the Drosophila ovary [31, 32, 40, 46, 47], this study is unique by virtue of focusing on the FSC region and utilizing the most current functional understanding of FSC numbers, locations, and behavior [2, 13].

## Methods

### Single-cell suspension preparation and fluorescence-activated cell sorting (FACS)

Female flies of genotype *C587-GAL4 / yw; UAS-CD8-RFP/* + were selected on the day of eclosion and maintained on rich food at 25 °C together with males for 4–7 days. More than 200 pairs of ovaries were dissected in ice-cold DPBS (Dulbecco’s phosphate-buffered saline) solution, and then gently washed twice by DPBS. The ovaries were digested in 700 µl solution containing 0.5% trypsin (Sigma, #T4799) and 0.25% collagenase (Invitrogen, #17,018–029) at 25 °C for about 20 min with gentle shaking. The dissociated cells were filtered through a 40-micron cell strainer (Falcon, #352,340), and then centrifuged at 400* g* at 4 °C for 5 min. Supernatant was removed and the cell pellet was resuspended in DPBS with 0.2% BSA. Cell suspensions were sorted using a FACS Aria III sorter MoFlo XDP (Beckman-Coulter) based on RFP signal. Cells from w^1118^ fly ovaries were used to set the negative fluorescence gate for the RFP panel. RFP-positive cells were sorted into DPBS containing 0.2% BSA, pelleted (400* g* for 10 min), and resuspended in DPBS. The LUNA-FL double fluorescent cell counter (Logos Biosystems) was used to count cell number and the live/dead cell ratio. The suspension of single cells, with > 85% live cells, had a final concentration of 500 cells/µl.

### Single-cell RNA sequencing (scRNA-seq)

The sorted cells were loaded on a Chromium Single cell Controller (10 X Genomics) using the Chromium Single-cell 3’ Library & Gel Bead kit v3 according to the manufacturer’s directions. Briefly, the Gel Beads containing the poly-T primer sequence were linked with a cell barcode and UMI (unique molecular identifier) and the cells are wrapped by “oil droplets” to form a GEM (gel bead in emulsion). Cells were lysed in these droplets and reverse-transcribed to form full-length cDNA sequence. After the oil droplets were broken and purified, the cDNA library was PCR amplified and ligated with sequencing primers. cDNA library quantification assays and quality check analysis were performed using an Agilent 2100 Bioanalyzer and Thermo Fisher Qubit Fluorometer. The library samples were then diluted to a 10 nM concentration and sequenced on an Illumina NovaSeq 6000 platform (Illumina, San Diego) for PE150 sequencing by Annoroad Gene Tech. Co., Ltd (Beijing, China). A total of 377,489,707 reads were obtained for the sample, with 290,154 mean reads per cell.

### Raw data preprocessing

The raw sequencing data was processed with alignment, barcode assignment, and UMI counting by Cell Ranger (version 6.1.1) using the previously published pipeline [79]. The reference index for Cell Ranger was built using the Drosophila melanogaster genome (version BDGP6.32) available on the Ensembl genome database.

### Single-cell RNA-seq data analysis

Cell Ranger’s raw gene count matrix was further analyzed using the Seurat (v4.0.2) R package with standard protocols [80]. Only genes expressed in more than 3 cells were kept, and only cells that have unique feature counts between 100 and 4500, and fewer than 20% mitochondrial counts (no cells were actually excluded due to high mitochondrial RNA counts) were selected for further analysis. Gene expression results were log-normalized, and then regressed on the percentage mitochondrial gene content. The top 16 principal components of principal component analysis (PCA) were used for clustering according to the Elbow Plot (resolution = 0.9). The ECs, FSC, and FCs were identified by markers of each cluster. t-SNE (t-distributed stochastic neighbor embedding) plots were used to visualize the clustering results. Mean expression values for each cluster were calculated by “AverageExpression” in Seurat. The R package “pheatmap” was used to generate heat-maps and the gene expression levels were scaled by row.

### MARCM and multicolor FSC lineages

In a typical MARCM (mosaic analysis with repressible cell marker) experiment, 1–3-day-old adult *Drosophila melanogaster* females with the appropriate genotype (*yw hs-Flp, UAS-nGFP, tub-GAL4 /yw; act-GAL80 FRT40A / FRT40A; act* > *CD2* > *GAL4/* + or *yw hs-Flp, UAS-nGFP, tub-GAL4 /yw; FRT42D act-GAL80 tub-GAL80 / FRT42D; act* > *CD2* > *GAL4/* +) were given a single 30-min heat shock at 37 °C. Afterwards, flies were incubated at 25 °C and maintained by frequent passage on normal rich food supplemented by fresh wet yeast before dissection 6 or 12 days later. For multicolor lineages, the procedure was similar but with multiple heat shocks [13] and the final genotype of *yw hs-flp / yw; tub-lacZ FRT40A FRT42B / ubi-GFP FRT40A FRT42B His2Av-mRFP*. Adult female ovaries were dissected in PBS, leaving the tip of the abdomen attached, and fixed in 4% paraformaldehyde (Electron Microscopy Sciences, #15,710) for 10 min at room temperature, covered to prevent bleaching of fluorophores. The ovaries were then washed three times with 1X PBST (PBS with 0.1% Triton X-100 and 0.05% Tween 20) and blocked at room temperature in blocking solution (PBST with 10% normal goat serum) for 30 min. Primary antibodies mouse anti-Fasciclin 3 (Developmental Studies Hybridoma Bank, #7G10, 1:250) and rabbit anti-GFP (Invitrogen, #A6455, 1:1000) were added and samples were nutated for an hour at room temperature. The ovaries were then rinsed with 1X PBST three times for 10 min each before being incubated with secondary antibodies Alexa Fluor-647 goat anti-mouse (Invitrogen, #A21236, 1:1000) and Alexa Fluor-488 goat anti-Rabbit (Invitrogen, #A11034, 1:1000) at room temperature for 1 h. The ovaries were then rinsed twice with PBST and once in PBS. Each ovary pair was then broken apart in PBS on a slide and mounted using *DAPI fluoromount-G* mounting medium (Southern BioTech, OB010020). Ovarioles were imaged with a Zeiss LSM700 or LSM800 confocal microscope, operated in part by the Zeiss ZEN software. Examples from single-color MARCM and multicolor lineage analyses are shown in Fig. 1.

### Protocol for identifying cell locations relative to landmark of anterior border of strong Fas3 staining

A variety of indicators help to ensure reproducible determination of the anterior border of strong Fas3 staining. These include germarium width, identifying the youngest stage 2b cyst and noting cell process locations. Starting with a mid-z-section, the youngest stage 2b cyst is identified by the criteria of spanning the germarium and not being at all rounded. Most germaria have only a single-candidate 2b cyst with a clearly more rounded stage 3 cyst more posterior. In such germaria, the Fas3 border runs along the posterior surface of the 2b cyst. In other germaria (up to about a quarter), a new 2b cyst has formed as a more posterior 2b cyst just starts to round. Here, the strong Fas3 border lies between the two 2b cysts. The strong Fas3 border can then be followed as a continuous surface through neighboring z-sections. Layer 1 cells immediately anterior to the Fas3 border have strong Fas3 staining on their posterior surface but weak or incomplete outlining of the anterior surface by Fas3. Labeling in MARCM clones usually allows visualization of FSC processes (even faintly when using a nuclear-targeted GFP marker). Layer 1 cell processes are present along the Fas3 border, whereas layer 2 processes are anterior to the stage 2b cyst. The widest part of the germarium is generally very close to the Fas3 border, with layer 1 FSCs at the widest location in about three quarters of samples and immediate FCs at that location in the rest.

### G-trace experiments

Flies of genotype *UAS-RFP, UAS-flp, ubi* > *stop* > *GFP / CyO; tub-GAL80(ts) / TM2* were crossed to (a) *tub-GAL80(ts) / CyO; fax-GAL4 / TM2* or (b) *Wnt4-GAL4 / CyO; tub-GAL80(ts) / TM2* or (c) *C587-GAL4; tub-GAL80(ts) / CyO; tub-GAL80(ts) / TM2* flies at 18 °C to collect experimental progeny with no balancer chromosomes and two (for *fax-GAL4* and *Wnt4-GAL4*) or three (for *C587-GAL4*) copies of *tub-GAL80(ts)*. Young (1–4 days old) experimental flies were then shifted to 29 or 30 °C for various periods and, in some cases, returned to 18 °C, as described in the “ Results” section for specific tests. For the final lineage tests, the conditions were 3 h at 30 °C followed by 13 h at 18 °C (*Wnt4-GAL4*), 6 h at 30 °C followed by 13 h at 18 °C (*C587GAL4*), or 10.5 h at 30 °C followed by 13 h at 18 °C (*fax-GAL4*) before dissecting for the first time-point, followed by a further 6 days at 18 °C before dissecting another cohort. An equivalent cohort of flies was kept at 18 °C throughout. In all cases, flies were transferred to fresh food every 2–3 days. Dissection, staining, mounting, and analyses were as described for MARCM experiments (RFP signals were visualized without antibody staining).

### Reagents used for determining gene expression patterns

Sema1-GFP (BL-60140, from Bloomington Drosophila Stock Center) is a Mi{MIC} insertion that results in an EGFP-tagged fusion protein. Plum-GFP is a protein fusion encoded by a genomic BAC transgene supplied by Dr. Oren Schuldiner [73]. CycB-GFP (BL-51568, from Bloomington Drosophila Stock Center) is a protein trap (GFP flanked by splice sites) transposon insertion into *cycB* that results in a GFP-tagged fusion protein. *Santa maria-GAL4* (BL-24521, from Bloomington Drosophila Stock Center) is a P-element insertion of a transgene that includes promoter sequences from − 2030 to + 30 of the *santa maria* gene upstream of yeast GAL4 coding sequence. Rabbit anti-Dystroglycan antibody was supplied by Dr. Stefan Baumgartner [72]. All samples were processed for immunocytochemistry as described above for MARCM lineages, with GFP or Dystroglycan primary antibodies visualized as described, or RFP protein from *santa maria-GAL4* > *UAS-RFP* visualized directly (without antibody staining), as in G-trace experiments.

## Supplementary Information

**Additional file 1: Figure S1.** Identity markers for peripheral groups in initial t-SNE clusters. Related to Figure 2.t-SNE plots of complete data setshowing color-coded relative expression levels of genes characteristic ofTF or cap cells,stalk or pre-stalk cellsorgermline cells.

**Additional File 2: Table 1 for Fig. 2C.** Cell barcodes. Cell barcodes for each dot on Fig. 2C.

**Additional File 3: Table 2 for Fig. 2E.** Expression characteristics for key cluster genes. Average expression values and percentage of cells with detected expression underlying the dot size and coloring in Fig. 2E.

**Additional File 4: Table 3.** Ranked list of genes characteristic of each cluster. Gene order for each cluster is determined by the ratio of expression in the characteristic cluster relative to average expression in all other cells. This enrichmentvalue for cluster “X” is calculated as E =/and denoted “avg_log2FC”. Also shown are the raw p values for significant differences in expression, adjusted p values after Bonferroni correction for multiple samples, the percentage of cells where the gene is detected in the characteristic clusterand in all other cells, as well as the normalized UMI counts for each cluster. Clusterscorrespond to Fig. 2C; each cluster is colored differently. This spreadsheet lists genes in each cluster down to an E value of 0.4. These genes and others with lower E values are listed in Additional File 5: Table 4.

**Additional File 5: Table 4.** Genes characteristic of each cluster. Genes characteristic of each cluster are listed together with information characteristic of Additional File 4: Table 1 in columns B-G and information characteristic of Additional File 8: Table 7 or Additional File 9: Table 8 in remaining columns. The threshold for inclusion in a cluster here is extremely lowcompared to Additional File 4: Table 3, to include 7547 genes.

**Additional File 6: Table 5 for Fig. 6A.** PC1 values. All genes listed by descending values for PC1.

**Additional File 7: Table 6 for Fig. 6B.** PC2 values. All genes listed by descending values for PC2.

**Additional File 8: Table 7.** Genes with increasing expression from ECs to FSCs and/or FSCs to FCs. Average normalized expression value for each cluster is listed in columns I-N for each groupand weighted values used to calculate expression among ECsand FCs. Fractional changes relative to the larger number were calculated for transitions from ECs to FSCs, FSCs to FCs, group 5 to group 1and group 1 to group 4. The resulting values in those four columns were classified into quartiles, with the first quartile colored dark brown for an increase or dark blue for a decrease, the second quartile colored light brown for an increase or light blue for a decrease, and the third quartile colored very light brown for an increase or very light blue for a decrease. Gene order was determined according to quartiles, first prioritizing genes that increased for both transitions, then genes increased only for the EC to FSC transition, listing first quartile genes first in each group. Horizontal green lines separate different categories and gene numbering begins at 1 for each category. Additional File 5: Table 4 lists all genes with latter columns in the same format as in this spreadsheet, regardless of the magnitude of changes from ECs to FSCs or FSCs to FCs.

**Additional File 9: Table 8.** Genes with decreasing expression from ECs to FSCs and/or FSCs to FCs. Average normalized expression value for each cluster is listed in columns I-N for each groupand weighted values used to calculate expression among ECsand FCs. Fractional changes relative to the larger number were calculated for transitions from ECs to FSCs, FSCs to FCs, group 5 to group 1and group 1 to group 4. The resulting values in those four columns were classified into quartiles, with the first quartile colored dark brown for an increase or dark blue for a decrease, the second quartile colored light brown for an increase or light blue for a decrease, and the third quartile colored very light brown for an increase or very light blue for a decrease. Gene order was determined according to quartiles, first prioritizing genes that decreased for both transitions, then genes decreased only for the EC to FSC transition, listing first quartile genes first in each group. Horizontal green lines separate different categories and gene numbering begins at 1 for each category. Additional File 5: Table 4 lists all genes with latter columns in the same format as in this spreadsheet, regardless of the magnitude of changes from ECs to FSCs or FSCs to FCs.

## Data Availability

The datasets generated and/or analyzed during the current study are available in the repository, Gene Expression Omnibus (GEO) database, https://www.ncbi.nlm.nih.gov/geo (accession number GEO: GSE208674). Additionally, a large fraction of the scRNA-seq data, organized in informative formats, is presented in Additional files 4, 5, 8 and 9.

## Abbreviations

FSC: Follicle stem cell
EC: Escort cell
r1 EC: Region 1 escort cell
r2a EC: Region 2a escort cell
FC: Follicle cell
AP: Anterior-posterior
DV: Dorsoventral
scRNA: Single-cell RNA
scRNA-seq: Single-cell RNA sequencing
Wnt: Wingless/Integrated
JAK-STAT: Janus Kinase-Signal Transducer and Activator of Transcription
TF: Terminal filament
GSC: Germline stem cell
Fas3: Fasciclin 3
FACS: Fluorescence-activated cell sorting
UMI: Unique molecular identifier
GEM: Gel bead in emulsion
PCA: Principal component analysis
MARCM: Mosaic analysis with repressible cell marker
IGS: Inner germarial sheath
*Eya*: *Eyes absent*
*Ilp6*: *Insulin-like peptide 6*
*croc*: *Crocodile*
*wun 2*: *wunen 2*
*bnb*: *Bangles and beads*
*Hf*: *Helical factor*
*Netrin A*: *NetA*
*Cre-loxP*: *Causes recombination-locus of crossing-over (x), P1*
*PCNA*: *Proliferating Cell Nuclear Antigen*
*Cas*: *Castor*
PI3K: Phospho-inositide 3’ kinase
Hh: Hedgehog
BMP: Bone morphogenetic protein
EGFR: Epidermal growth factor receptor
FGFR: Fibroblast growth factor receptor
*Sema 1a*: *Semaphorin 1a*
*PlexA*: *Plexin A*
GFP: Green fluorescent protein
RFP: Red fluorescent protein
ECM: Extracellular matrix
TGF-β: Transforming growth factor β
CycB: Cyclin B
t-SNE: t-distributed stochastic neighbor embedding
PBS: Phosphate-buffered saline

## References

[1] Clevers H, Watt FM (2018). Defining adult stem cells by function, not by phenotype. Annu Rev Biochem.

[2] Kalderon D. Investigating adult stem cells through lineage analyses. Stem Cell Rev Rep. 2021;18(1):2–22. 10.1007/s12015-021-10282-z PMC8799514 34677818

[3] Post Y, Clevers H (2019). Defining adult stem cell function at its simplest: the ability to replace lost cells through mitosis. Cell Stem Cell.

[4] Chatzeli L, Simons BD. Tracing the dynamics of stem cell fate. Cold Spring Harbor Perspect Biol. 2020;12(6):a036202. 10.1101/cshperspect.a036202 PMC7263083 31932319

[5] Dray N, Than-Trong E, Bally-Cuif L (2021). Neural stem cell pools in the vertebrate adult brain: Homeostasis from cell-autonomous decisions or community rules?. BioEssays.

[6] Goodell MA, Nguyen H, Shroyer N (2015). Somatic stem cell heterogeneity: diversity in the blood, skin and intestinal stem cell compartments. Nat Rev Mol Cell Biol.

[7] Jones PH (2010). Stem cell fate in proliferating tissues: equal odds in a game of chance. Dev Cell.

[8] Stine RR, Matunis EL (2013). Stem cell competition: finding balance in the niche. Trends Cell Biol.

[9] Reilein A, Melamed D, Tavare S, Kalderon D (2018). Division-independent differentiation mandates proliferative competition among stem cells. Proc Natl Acad Sci U S A.

[10] Ritsma L, Ellenbroek SI, Zomer A, Snippert HJ, de Sauvage FJ, Simons BD (2014). Intestinal crypt homeostasis revealed at single-stem-cell level by in vivo live imaging. Nature.

[11] Rompolas P, Mesa KR, Kawaguchi K, Park S, Gonzalez D, Brown S (2016). Spatiotemporal coordination of stem cell commitment during epidermal homeostasis. Science.

[12] Greulich P, Simons BD (2016). Dynamic heterogeneity as a strategy of stem cell self-renewal. Proc Natl Acad Sci U S A.

[13] Reilein A, Melamed D, Park KS, Berg A, Cimetta E, Tandon N (2017). Alternative direct stem cell derivatives defined by stem cell location and graded Wnt signalling. Nat Cell Biol.

[14] Beumer J, Clevers H. Cell fate specification and differentiation in the adult mammalian intestine. Nat Rev Mol Cell Biol. 2020;22(1):39–53. 10.1038/s41580-020-0278-0 32958874

[15] Donati G, Watt FM (2015). Stem cell heterogeneity and plasticity in epithelia. Cell Stem Cell.

[16] Greenspan LJ, de Cuevas M, Le KH, Viveiros JM, Matunis EL. Activation of the EGFR/MAPK pathway drives transdifferentiation of quiescent niche cells to stem cells in the Drosophila testis niche. Elife. 2022;11:e70810. 10.7554/eLife.70810 PMC9038189 35468055

[17] Melamed D, Kalderon D. Opposing JAK-STAT and Wnt signaling gradients define a stem cell domain by regulating differentiation at two borders. Elife. 2020;9:e61204. 10.7554/eLife.61204 PMC7695452 33135631

[18] Rust K, Nystul T (2020). Signal transduction in the early Drosophila follicle stem cell lineage. Curr Opin Insect Sci.

[19] Bastock R, St JD (2008). Drosophila oogenesis. Curr Biol.

[20] Eliazer S, Buszczak M (2011). Finding a niche: studies from the Drosophila ovary. Stem Cell Res Ther.

[21] Spradling A, Fuller MT, Braun RE, Yoshida S (2011). Germline stem cells. Cold Spring Harb Perspect Biol.

[22] Decotto E, Spradling AC (2005). The Drosophila ovarian and testis stem cell niches: similar somatic stem cells and signals. Dev Cell.

[23] Kirilly D, Wang S, Xie T (2011). Self-maintained escort cells form a germline stem cell differentiation niche. Development.

[24] Dai W, Peterson A, Kenney T, Burrous H, Montell DJ (2017). Quantitative microscopy of the Drosophila ovary shows multiple niche signals specify progenitor cell fate. Nat Commun.

[25] Margolis J, Spradling A (1995). Identification and behavior of epithelial stem cells in the Drosophila ovary. Development.

[26] Tworoger M, Larkin MK, Bryant Z, Ruohola-Baker H (1999). Mosaic analysis in the drosophila ovary reveals a common hedgehog-inducible precursor stage for stalk and polar cells. Genetics.

[27] Duhart JC, Parsons TT, Raftery LA (2017). The repertoire of epithelial morphogenesis on display: Progressive elaboration of Drosophila egg structure. Mech Dev.

[28] Riechmann V, Ephrussi A (2001). Axis formation during Drosophila oogenesis. Curr Opin Genet Dev.

[29] Zhang Y, Kalderon D (2001). Hedgehog acts as a somatic stem cell factor in the Drosophila ovary. Nature.

[30] Melamed D, Choi A, Reilein A, Kalderon D. Spatial regulation of Drosophila ovarian Follicle Stem Cell division rates and cell cycle transitions. bioRxiv. 2022. 10.1101/2022.06.22.497017. 10.1371/journal.pgen.1010965 PMC10553835 37747936

[31] Jevitt A, Chatterjee D, Xie G, Wang XF, Otwell T, Huang YC (2020). A single-cell atlas of adult Drosophila ovary identifies transcriptional programs and somatic cell lineage regulating oogenesis. PLoS Biol.

[32] Slaidina M, Gupta S, Banisch TU, Lehmann R (2021). A single-cell atlas reveals unanticipated cell type complexity in Drosophila ovaries. Genome Res.

[33] Forbes AJ, Spradling AC, Ingham PW, Lin H (1996). The role of segment polarity genes during early oogenesis in Drosophila. Development.

[34] Godt D, Laski FA (1995). Mechanisms of cell rearrangement and cell recruitment in Drosophila ovary morphogenesis and the requirement of bric a brac. Development.

[35] Xie T, Spradling AC (1998). decapentaplegic is essential for the maintenance and division of germline stem cells in the Drosophila ovary. Cell.

[36] Allbee AW, Rincon-Limas DE, Biteau B. Lmx1a is required for the development of the ovarian stem cell niche in Drosophila. Development. 2018;145(8):dev163394. 10.1242/dev.163394 PMC5964653 29615466

[37] Buszczak M, Paterno S, Lighthouse D, Bachman J, Planck J, Owen S (2007). The carnegie protein trap library: a versatile tool for Drosophila developmental studies. Genetics.

[38] Vied C, Reilein A, Field NS, Kalderon D (2012). Regulation of stem cells by intersecting gradients of long-range niche signals. Dev Cell.

[39] Waghmare I, Page-McCaw A. Wnt signaling in stem cell maintenance and differentiation in the Drosophila Germarium. Genes (Basel). 2018;9(3):127. 10.3390/genes9030127 PMC5867848 29495453

[40] Tu R, Duan B, Song X, Chen S, Scott A, Hall K (2021). Multiple niche compartments orchestrate stepwise germline stem cell progeny differentiation. Curr Biol.

[41] McGregor JR, Xi R, Harrison DA (2002). JAK signaling is somatically required for follicle cell differentiation in Drosophila. Development.

[42] Reilein A, Kogan HV, Misner R, Park KS, Kalderon D. Adult stem cells and niche cells segregate gradually from common precursors that build the adult Drosophila ovary during pupal development. Elife. 2021;10:e69749. 10.7554/eLife.69749 PMC8536258 34590579

[43] Wang X, Page-McCaw A. Wnt6 maintains anterior escort cells as an integral component of the germline stem cell niche. Development. 2018;145(3):dev158527. 10.1242/dev.158527 PMC5818006 29361569

[44] Kretzschmar K, Watt FM (2012). Lineage tracing. Cell.

[45] Evans CJ, Olson JM, Ngo KT, Kim E, Lee NE, Kuoy E (2009). G-TRACE: rapid Gal4-based cell lineage analysis in Drosophila. Nat Methods.

[46] Rust K, Byrnes LE, Yu KS, Park JS, Sneddon JB, Tward AD (2020). A single-cell atlas and lineage analysis of the adult Drosophila ovary. Nat Commun.

[47] Shi J, Jin Z, Yu Y, Zhang Y, Yang F, Huang H (2021). A progressive somatic cell niche regulates germline cyst differentiation in the drosophila ovary. Curr Biol..

[48] Chung S, Le TP, Vishwakarma V, Cheng YL, Andrew DJ. Isoform-specific roles of the Drosophila filamin-type protein Jitterbug (Jbug) during development. Genetics. 2021;219(2):iyab100. 10.1093/genetics/iyab100 PMC8860385 34173831

[49] Wang X, Page-McCaw A (2014). A matrix metalloproteinase mediates long-distance attenuation of stem cell proliferation. J Cell Biol.

[50] Sun J, Deng WM (2005). Notch-dependent downregulation of the homeodomain gene cut is required for the mitotic cycle/endocycle switch and cell differentiation in Drosophila follicle cells. Development.

[51] Wang ZA, Huang J, Kalderon D (2012). Drosophila follicle stem cells are regulated by proliferation and niche adhesion as well as mitochondria and ROS. Nat Commun.

[52] Song X, Xie T (2002). DE-cadherin-mediated cell adhesion is essential for maintaining somatic stem cells in the Drosophila ovary. Proc Natl Acad Sci USA.

[53] Hartman TR, Zinshteyn D, Schofield HK, Nicolas E, Okada A, O'Reilly AM (2010). Drosophila Boi limits Hedgehog levels to suppress follicle stem cell proliferation. J Cell Biol.

[54] Huang J, Kalderon D (2014). Coupling of Hedgehog and Hippo pathways promotes stem cell maintenance by stimulating proliferation. J Cell Biol.

[55] O'Reilly AM, Lee HH, Simon MA (2008). Integrins control the positioning and proliferation of follicle stem cells in the Drosophila ovary. J Cell Biol.

[56] Herrera SC, Sainz de la Maza D, Grmai L, Margolis S, Plessel R, Burel M, et al. Proliferative stem cells maintain quiescence of their niche by secreting the Activin inhibitor Follistatin. Dev Cell. 2021;56(16):2284–94. 10.1016/j.devcel.2021.07.010 PMC8387025 34363758

[57] Upadhyay M, Kuna M, Tudor S, Martino Cortez Y, Rangan P (2018). A switch in the mode of Wnt signaling orchestrates the formation of germline stem cell differentiation niche in Drosophila. PLoS Genet.

[58] Harris RE, Ashe HL (2011). Cease and desist: modulating short-range Dpp signalling in the stem-cell niche. EMBO Rep.

[59] Ingham PW (2022). Hedgehog signaling. Curr Top Dev Biol.

[60] Forbes AJ, Lin H, Ingham PW, Spradling AC (1996). hedgehog is required for the proliferation and specification of ovarian somatic cells prior to egg chamber formation in Drosophila. Development.

[61] Sahai-Hernandez P, Nystul TG (2013). A dynamic population of stromal cells contributes to the follicle stem cell niche in the Drosophila ovary. Development.

[62] Kakugawa S, Langton PF, Zebisch M, Howell S, Chang TH, Liu Y (2015). Notum deacylates Wnt proteins to suppress signalling activity. Nature.

[63] Zeng W, Wharton KA, Mack JA, Wang K, Gadbaw M, Suyama K (2000). naked cuticle encodes an inducible antagonist of Wnt signalling. Nature.

[64] Matsuoka S, Gupta S, Suzuki E, Hiromi Y, Asaoka M (2014). gone early, a novel germline factor, ensures the proper size of the stem cell precursor pool in the Drosophila ovary. PLoS ONE.

[65] Sapir A, Schweitzer R, Shilo BZ (1998). Sequential activation of the EGF receptor pathway during Drosophila oogenesis establishes the dorsoventral axis. Development.

[66] Baeg GH, Lin X, Khare N, Baumgartner S, Perrimon N (2001). Heparan sulfate proteoglycans are critical for the organization of the extracellular distribution of Wingless. Development.

[67] Avanesov A, Blair SS (2013). The Drosophila WIF1 homolog Shifted maintains glypican-independent Hedgehog signaling and interacts with the Hedgehog co-receptors Ihog and Boi. Development.

[68] Han C, Belenkaya TY, Wang B, Lin X (2004). Drosophila glypicans control the cell-to-cell movement of Hedgehog by a dynamin-independent process. Development.

[69] Yu L, Zhou Y, Cheng S, Rao Y (2010). Plexin a-semaphorin-1a reverse signaling regulates photoreceptor axon guidance in Drosophila. J Neurosci.

[70] Stedden CG, Menegas W, Zajac AL, Williams AM, Cheng S, Ozkan E (2019). Planar-polarized semaphorin-5c and plexin A promote the collective migration of epithelial cells in drosophila. Curr Biol..

[71] Cerqueira Campos F, Dennis C, Alegot H, Fritsch C, Isabella A, Pouchin P, et al. Oriented basement membrane fibrils provide a memory for F-actin planar polarization via the Dystrophin-Dystroglycan complex during tissue elongation. Development. 2020;147(7):dev186957. 10.1242/dev.186957 PMC7157587 32156755

[72] Schneider M, Khalil AA, Poulton J, Castillejo-Lopez C, Egger-Adam D, Wodarz A (2006). Perlecan and Dystroglycan act at the basal side of the Drosophila follicular epithelium to maintain epithelial organization. Development.

[73] Yu XM, Gutman I, Mosca TJ, Iram T, Ozkan E, Garcia KC (2013). Plum, an immunoglobulin superfamily protein, regulates axon pruning by facilitating TGF-beta signaling. Neuron.

[74] Zielke N, Korzelius J, van Straaten M, Bender K, Schuhknecht GF, Dutta D (2014). Fly-FUCCI: a versatile tool for studying cell proliferation in complex tissues. Cell Rep.

[75] Wang X, Wang T, Jiao Y, von Lintig J, Montell C (2010). Requirement for an enzymatic visual cycle in Drosophila. Curr Biol.

[76] Fadiga J, Nystul TG. The follicle epithelium in the Drosophila ovary is maintained by a small number of stem cells. Elife. 2019;8:e49050. 10.7554/eLife.49050 PMC6946398 31850843

[77] Kalderon D, Melamed D, Reilein A. Follicle Stem Cells (FSCs) in the Drosophila ovary; a critique of published studies defining the number, location and behavior of FSCs. bioRxiv. 2020:2020.06.25.171579. 10.1101/2020.06.25.171579.

[78] Kalderon D, Melamed D, Reilein A. The number of follicle stem cells in a Drosophila ovariole. bioRxiv. 2021:2021.10.25.465475. 10.1101/2021.10.25.465475.

[79] Zheng GX, Terry JM, Belgrader P, Ryvkin P, Bent ZW, Wilson R (2017). Massively parallel digital transcriptional profiling of single cells. Nat Commun.

[80] Hao Y, Hao S, Andersen-Nissen E, Mauck WM, Zheng S, Butler A (2021). Integrated analysis of multimodal single-cell data. Cell..

