# Supplementary material for: Single-cell expression profile of Drosophila ovarian follicle stem cells illuminates spatial differentiation in the germarium
Source: BMC Biol. 2023 Jun 20;21:143. doi: 10.1186/s12915-023-01636-9 (PMC10283321; doi:10.1186/s12915-023-01636-9)
Supplement: Supplementary file 1 — Additional file 1: Figure S1. Identity markers for peripheral groups in initial t-SNE clusters. Related to Figure 2.t-SNE plots of complete data setshowing color-coded relative expression levels of genes characteristic ofTF or cap cells,stalk or pre-stalk cellsorgermline cells. [file 12915_2023_1636_MOESM1_ESM.pdf]

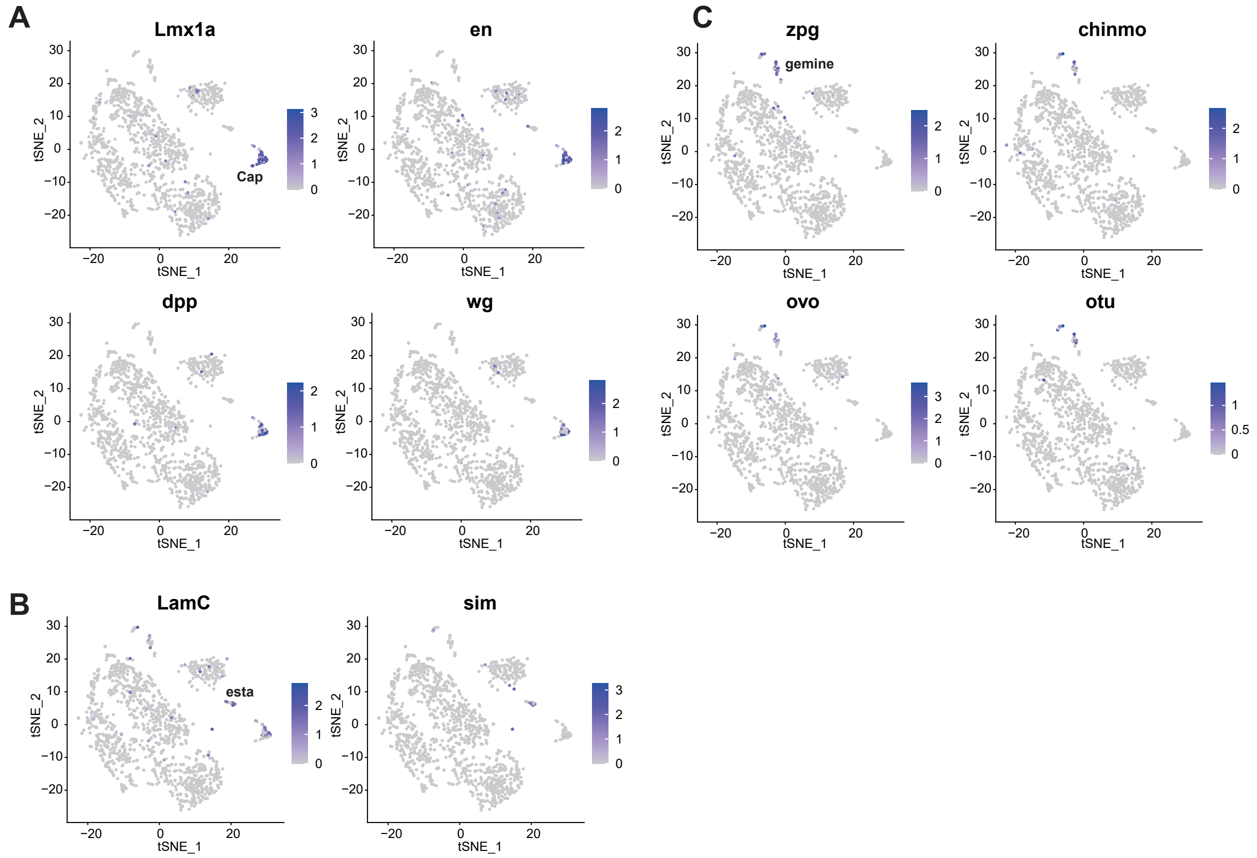

Additional File 1: Figure S1. (Additional File 1 Fig S1.pdf)

Identity markers for peripheral groups in initial t-SNE clusters. Related to Figure 2.

(A-C) t-SNE plots of complete data set (Fig. 2A) showing color-coded relative expression levels of genes characteristic of (A) TF or cap cells (*Lmx1a*, *en*, *dpp*, *wg*), (B) stalk or pre-stalk cells (*LamC*, *sim*) or (C) germline cells (*zpg*, *chinmo*, *ovo* and *otu*).
